# COVID-19 heterogeneity in islands chain environment

**DOI:** 10.1371/journal.pone.0263866

**Published:** 2022-05-18

**Authors:** Monique Chyba, Prateek Kunwar, Yuriy Mileyko, Alan Tong, Winnie Lau, Alice Koniges

**Affiliations:** 1 Department of Mathematics, University of Hawai‘i at Manoa Department of Mathematics, Honolulu, Hawai‘i, United States of America; 2 Hawai‘i Data Science Institute, University of Hawai‘i at Manoa, Honolulu, Hawai‘i, United States of America; Texas A&M University College Station, UNITED STATES

## Abstract

**Background:**

It is critical to capture data and modeling from the COVID-19 pandemic to understand as much as possible and prepare for future epidemics and possible pandemics. The Hawaiian Islands provide a unique opportunity to study heterogeneity and demographics in a controlled environment due to the geographically closed borders and mostly uniform pandemic-induced governmental controls and restrictions.

**Objective:**

The goal of the paper is to quantify the differences and similarities in the spread of COVID-19 among different Hawaiian islands as well as several other archipelago and islands, which could potentially help us better understand the effect of differences in social behavior and various mitigation measures. The approach should be robust with respect to the unavoidable differences in time, as the arrival of the virus and promptness of mitigation measures may vary significantly among the chosen locations. At the same time, the comparison should be able to capture differences in the overall pandemic experience.

**Methods:**

We examine available data on the daily cases, positivity rates, mobility, and employ a compartmentalized model fitted to the daily cases to develop appropriate comparison approaches. In particular, we focus on merge trees for the daily cases, normalized positivity rates, and *baseline transmission rates* of the models.

**Results:**

We observe noticeable differences among different Hawaiian counties and interesting similarities between some Hawaiian counties and other geographic locations. The results suggest that mitigation measures should be more localized, that is, targeting the county level rather than the state level if the counties are reasonably insulated from one another. We also notice that the spread of the disease is very sensitive to unexpected events and certain changes in mitigation measures.

**Conclusions:**

Despite being a part of the same archipelago and having similar protocols for mitigation measures, different Hawaiian counties exhibit quantifiably different dynamics of the spread of the disease. One potential explanation is that not sufficiently targeted mitigation measures are incapable of handling unexpected, localized outbreak events. At a larger-scale view of the general spread of the disease on the Hawaiian island counties, we find very interesting similarities between individual Hawaiian islands and other archipelago and islands.

## Introduction

Significant local variations in the spread of COVID-19 have been established in heterogeneous environments. For example, Thomas, et al., compares nineteen different cities and counties in the US [1]. They found that small differences in network models for interdependence and social interaction as well as the effects due to uneven population distributions can lead to substantial differences in infection timing and severity, leading different areas in each city to have vastly different experiences of the pandemic. Similar patterns associated with heterogeneity have been made for entire nations, such as the work comparing the most affected cities in China [2]. These works are based on the premise that substantial heterogeneity in social relationships at various scales affect the viral spread. It is unclear, however, whether or not such heterogeneity is a critical factor for an island chain and such study is absent from the literature. This is of utmost importance due to islands’ vulnerability to any pandemic, especially for native populations as demonstrated for example with the introduction of measles to the Pacific Islands in the 1800’s [3]. Islands are smaller contained populations, and thus epidemiological models may require adjustments to properly apply them to disease containment strategies. Identifying if major local variations can be expected for an island chain in the spread of COVID-19 is crucial since it directly impacts the effectiveness of mitigation measures, vaccine distribution, and health-care management. We focus here on a specific island chain, the Hawaiian archipelago, and take a somewhat different approach by comparing pandemic dynamics within individual islands and identifying countries or geo-regions exhibiting similar properties.

**Fig 1:**
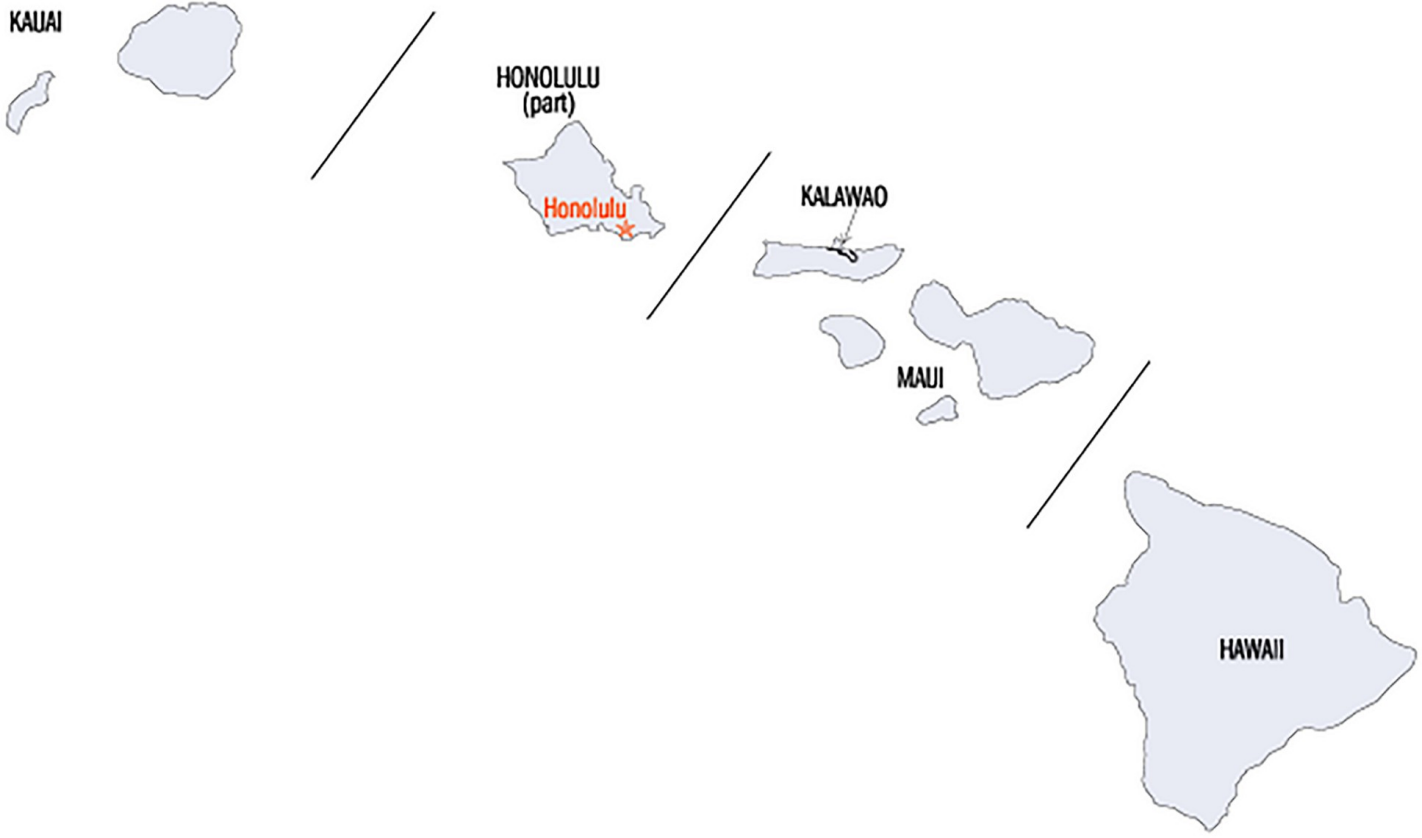
The state of Hawai‘i and its counties. Kaua‘i county encompasses the islands of Kaua‘i and Ni’ihau. Honolulu county contains only the island of Oahu. Maui county comprises the islands of Maui, Moloka’i, Lana’i, and Kaho’olawe. Hawai‘i county contains only the island of Hawai‘i. Taken from Maps of World, Countries & Cities—Mapsof.Net.

The Hawaiian Islands are an archipelago of eight major islands, with only seven of them being populated. The State is divided into five counties: Hawai‘i, Honolulu, Kalawao, Kaua‘i, and Maui. Since Kalawao is the smallest county in all of the 50 states in terms of both population and land area, we focus here on only the four major counties (Fig 1). Honolulu city and county is the most populated county of the state, with 69% of the state’s population. Hawai‘i county has the largest land mass of 63% of the entire state, but comes second in resident population. Third by population is Maui county, which spans the islands of Maui, Moloka‘i, Lanai, and Kaho‘olawe. Kaua‘i county, which spans the islands Kaua‘i and Ni‘ihau, has the smallest population of the four counties studied in this paper (S1 Table). Demographics information about the four counties can be found in S1 Fig. The majority of health facilities are located in Honolulu county. More precisely, each of the neighbor islands has at least one hospital, but some like the one on Lanai are severely limited in services. Honolulu County has 16 hospital branches, Maui county has 4, Kauai county has 3 and Hawai‘i county has 6, but these vary in size, services and facilities, with most being quite limited compared to Mainland hospitals.

The Hawaiian Islands are at close proximity to each other and it is relatively quick and easy to travel between them. (Pandemic-related imposed restrictions made inter-island travel more difficult, however.) The six major islands are connected by more than dozens of flights everyday. Oahu is the hub of Hawaii inter-island travel. The most frequent services are between the islands of Oahu, Maui, Kauai and the Big Island. Daily smaller planes also exists to connect to the islands of Lanai and Molokai. Accounting for all types of travel (international and domestic) before the pandemic, there was an average of 30,000 daily passenger counts. When the COVID-19 pandemic first started, inter-island travel halted due to a mandatory 14-day quarantine in addition to the shutdown of activity. Travel later picked up again in October 15, 2020 with a travel program.

Our goal is to demonstrate that islands in general, whether they belong to the same archipelago or not, respond differently to the pandemic and cannot be aggregated into one single class. This is possibly due to the inherent stochasticity and non-linearity of viral spread which gets exacerbated by the small population of the islands.

## Materials and methods

### Data sources

For the Hawaiian Islands, we compiled reported COVID-19 cases per day from March 6, 2020 to January 15, 2021. Specifically, we collected the numbers of COVID-19 cases for the four counties under study from various dashboards. In addition to comparison between the 4 Hawaiian counties, we also collected and compared the Hawaiian counties to other geographic locations with similar isolation features. See S1 Appendix for a list of data sources used for the COVID-19 daily cases. For each county, we also compiled the distribution of cases per zip code. The zip code tabulation areas can be found from the State of Hawai‘i Office of Planning 2010 Census Reference Maps. Since those numbers are not compiled in any open source spreadsheet, they need to be fetched from the Disease Outbreak Control Division Dashboard under their Hawai‘i COVID-19 maps daily. To obtain mobility data we used the open source SafeGraph COVID-19 Data Consortium [4] that provides social distancing metrics illustrating the daily view of movement between census block groups.

**Fig 2:**
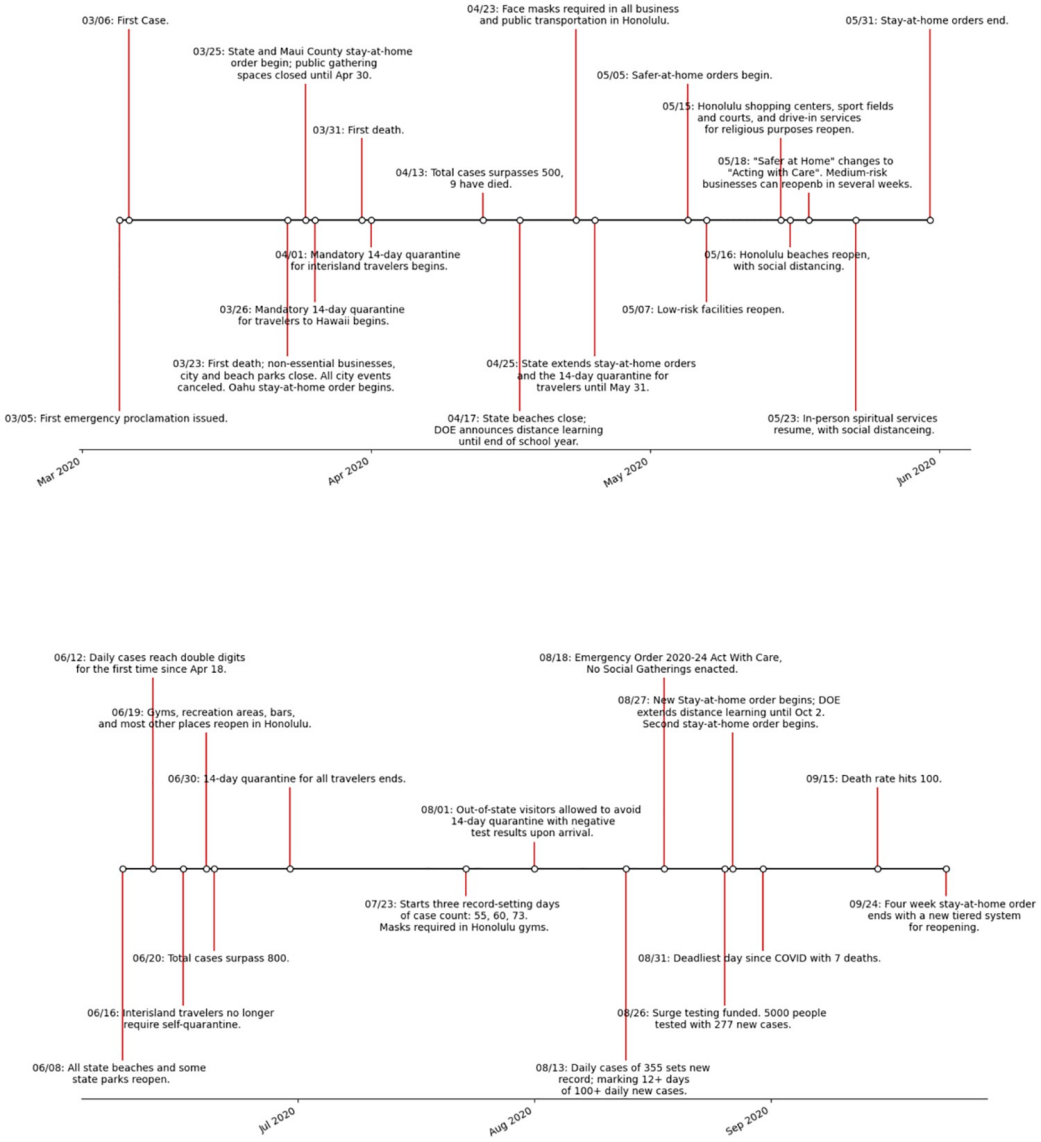
Hawai‘i Covid-19 mitigation timeline. Timeline of events related to the pandemic in the State of Hawai‘i from March 6, 2020 to September 24, 2020.

**Fig 3:**
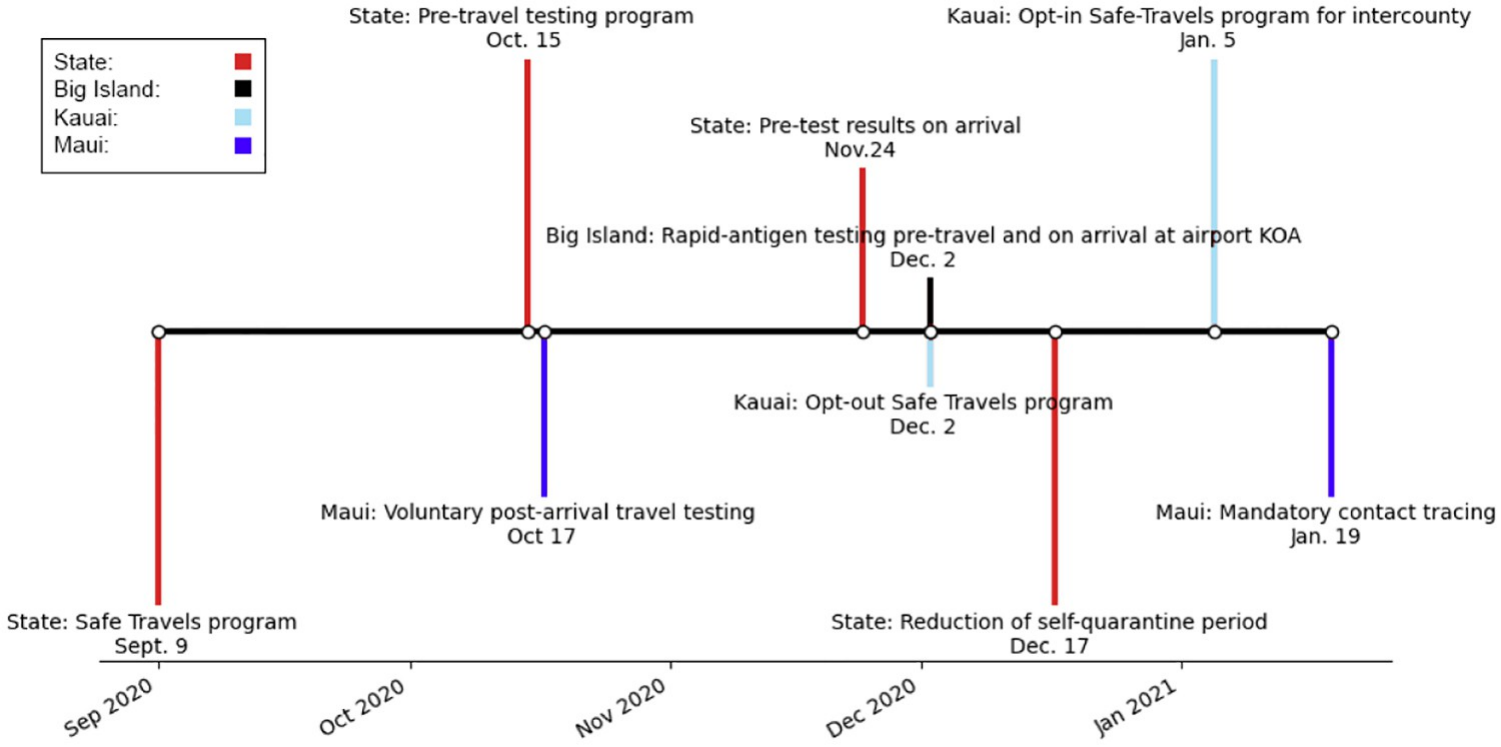
Safe travel protocols per counties. Kaua‘i county has the most restricted travel regulations since Dec. 2, 2020 following a significant initial surge in cases with the introduction of the Safe Travels Program on October 15, 2020.

The transmission rate in our model is optimized to reflect non pharmaceutical mitigation interventions. Fig 2 displays the timeline from March 6 to September 24. The primary events assumed to impact the epidemiological curves after September 24 are noted in the timeline. The first of these is the *safe travel program* implemented by the State of Hawai‘i in Fig 3. In addition, the entire state has moved from Tier 1 to Tier 2 on October 22, 2020 (which are metrics for reopening with Tier 2 employing lighter mitigation measures) and has stayed in that phase throughout the period studied in this paper. Note also that the State of Hawai‘i started administering vaccines on Dec 15, 2020. As of January 17, 2021 the State of Hawai‘i recorded 76,498 administered vaccines doses. Of additional note are the deadliest day since September 24, and the global maximum happened on October 14, 2020 with a count of 14 individuals.

Travel to the Hawaiian islands has been heavily mandated by the *Safe Travels Program*. With few exceptions, for travel to the islands prior to October 15, 2020, all travelers are expected to self-quarantine for two weeks upon arrival, regardless of residency status. This includes interisland travel. Starting on October 15, 2020 a way to bypass this quarantine was implemented. One may take a Nucleic Acid Amplification Test up to 72 hours before the originating flight, and it must return a negative result in order to avoid quarantine. Kauai opted out of the testing option and maintained mandatory quarantine. In January, Maui county implemented mandatory contact tracing through the AlohaSafe Alert App. (https://hawaiicovid19.com/travel) for all incoming travelers.

### Comparing to other locations

We also compare our results to three geographical locations with some similar water-type geographical boundaries, namely Iceland, Japan, and Puerto Rico. Japan is natural to consider given the close relationship it has with Hawai‘i, both in terms of travel destination and ethnicity. Japan, while formed of many islands, is primarily divided into five major islands. The most populated is Honshu, and it is well connected to Hokkaido, Kyushu and Shikoku, with people able to travel by car, train, ferry or plane. Okinawa is isolated and accessible only by plane. However, by the end of 2020 the cumulative daily cases for Okinawa were less than 2% of Japan’s total case number. Prior to Jan 15, 2021 the daily cases for Japan are dominated by case numbers in the prefecture of Honshu. Unfortunately, prefecture specific data are difficult to access, and since our analysis is qualitative we opted to consider Japan data as reflective of the Island of Honshu. Iceland was chosen for its stringent travel restrictions comparable to the one of the State of Hawai‘i before the safe travel program. The US territory of Puerto-Rico shares climate similarity with Hawai‘i and therefore was also a natural choice for comparison.

### Assumptions on travel

We have used several of these critical dates and restrictions to adjust incoming travelers and their transmission parameters in our modeling. Regarding travel flux for the State of Hawai‘i since the implementation of the Safe Travel Program on October 15, 2020, we compiled data from the COVID-19 State of Hawai‘i Travel portal [5]. Based on the Safe Travels Digital Platform from the State of Hawai‘i, we are assuming a pre-travel testing rate of 86%, and a false negative rate of 0.5%. We also assume 1% of untested visitors go into exposed isolation (namely we removed an assumed number of exempt travelers) and a 5% prevalence for the virus. The pre-testing rates for travelers to Maui county is higher, and assumed to be 95%. Traveler average influx is modeled as a piece-wise linear function over two week intervals between October 15, 2020 and January 15, 2021.

The average assumed influx for Iceland, Japan and the US territory Puerto Rico is simplified to be linear over the same time period. See S2 Table for the estimated values.

### Testing and test positivity

We created plots to study the correlation between daily cases, testing, and test positivity (i.e., the percent of tests for COVID-19 that came back positive) for each county. To create the overlayed plots, the metrics are normalized by calculating each data point as a percent of the maximum of the corresponding metric over the whole observation period and using a 7-day rolling average.

### Computational method

There are two main classes of epidemiological models for this type of disease spread: compartmental models [6–8] and agent-based models [9–12]. In this paper, we use a compartmentalized model inspired by [13], which is based on a standard discrete SEIR model. A key extension to a standard SEIR model that we have added for this paper is a new compartmental group for travelers. Indeed, the tourist population plays a prominent role in Hawai‘i and due to our isolated geographic location we are able to to collect precise information about daily arrivals and departures. On some islands there are routinely more tourists than residents, and the tourists typically stay only a week or two and are thus better cast as travelers with their own demographics.

In our model, a given population is divided into four compartments: Susceptible (not currently infected), Exposed (infected with no symptoms), Infected (infected with symptoms), and Removed (recovered or deceased). Moreover, we subdivide the entire population into three additional disjoint groups: the general community (C), healthcare workers (H) and visitors (V). Healthcare workers are in a separate compartment since they appear to play a more unique role in the spread of diseases, especially for Covid-19. This includes potentially different exposure to the disease (both through exposure and mitigational personal protective equipment) as well as potentially taking more precautionary measures than typical individuals [13–15]. In practice, this is incorporated into the modeling by assigning slightly different transmission parameters to the compartment containing the health care workers.

**Fig 4:**
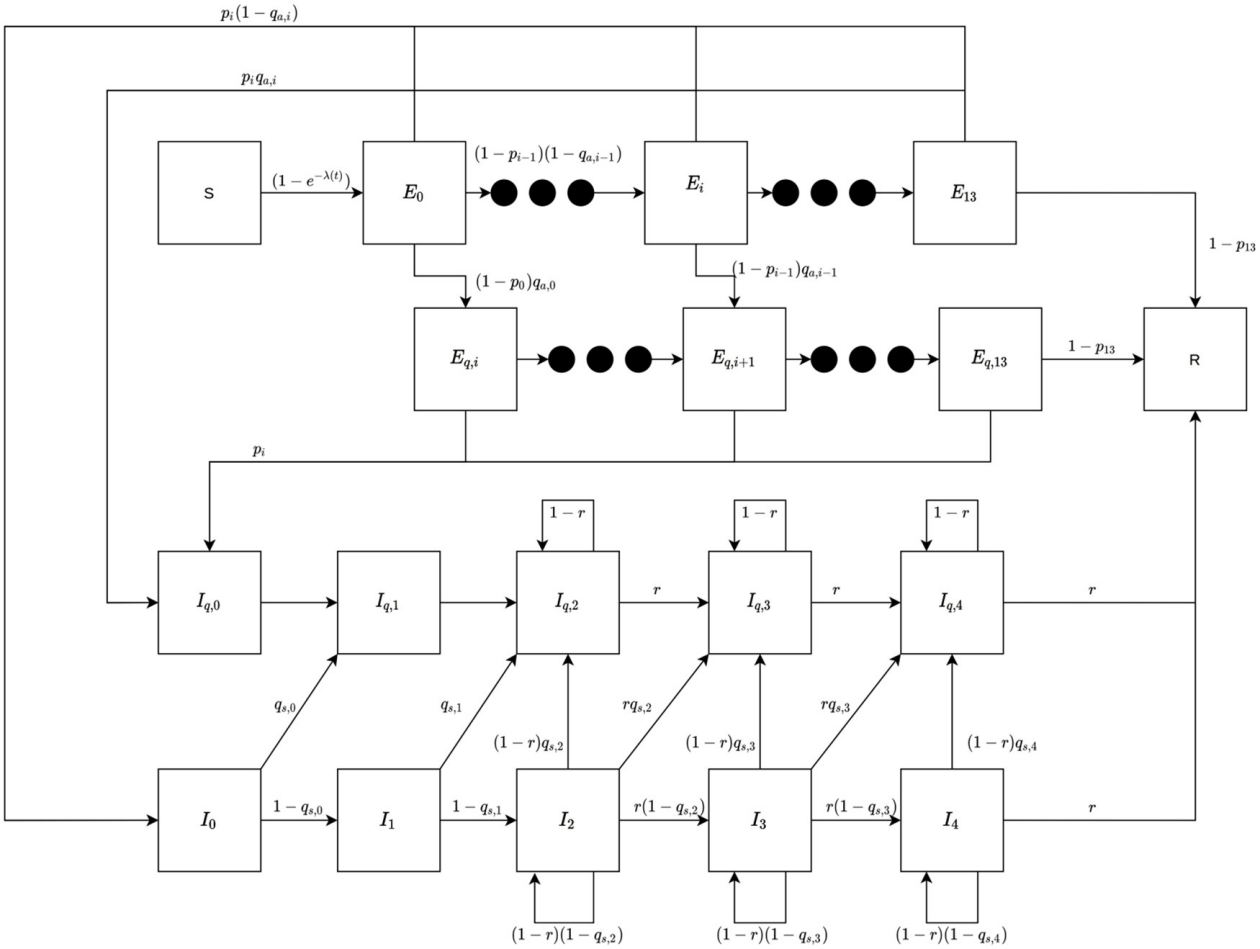
Diagram of our basic compartmental model. Illustration of the compartments and their interactions.

Visitors, who are only considered after October 15 (when the safe travels Hawai‘i program began) are further broken down into two categories: returning residents and tourists. While the returning residents are absorbed into the community compartment, the tourists are treated as a separate compartment. In addition, the sub-compartments denoted Exposed and Infected (in each population group) are split into multiple stages each day to better reflect the progression of the disease. There are two key dynamics of each population group: the dynamics of Susceptible individuals and the dynamics of the rest of the compartments. The time dependent *hazard rate*, λ(*t*), governs the susceptible dynamics. It reflects the probability, 1 − *e*^−λ(*t*)^, of an individual becoming exposed at time *t*. The hazard rate is different for different population groups and takes into account interactions between the groups, thus coupling their dynamics. For more information regarding dynamics equations of the model, see S2 Appendix, and Fig 4 which provides a diagram of the dynamics within each compartment.

Key to controlling the spread of the disease within our model is the parameter *β* that models the basal transmission rate due to various interactions among individuals. Our model optimizes *β* to fit daily cases for a specific geographic location. We use several different values of *β* to capture changes in COVID-19 mitigation policy based on the location. Table 1 displays the variables and parameters common to all simulations in this paper. (Optimized *β*’s are given in the Results section.) We introduce the parameters *p*_*i*_ as the probability to develop symptoms on day *i*, and choose them such that if symptoms do develop, it takes between 2 to 14 days, with a mean between 4 and 6 days [16], while assuming that about 40% of all infections remain asymptomatic. The values of *q*_*s*,*i*_ reflect the sentiment that symptomatic individuals are likely to isolate, especially after a couple of days of symptoms. In addition, the parameter *r* is the probability of transitioning from one stage of the illness to the next (with the final stage being recovery or death). Based on prior work [17], we choose *r* to yield an expected length of illness of 17 days. Hospitals took precautions and implemented guidance for their healthcare workers by taking drastic measures to guarantee the safety of their healthcare workers as well as patients which is reflected through the parameter *κ* [18]. Note that the hospitals in Hawaii did not run out of protective gear due to the fact the numbers of cases stayed relatively low compare to other States. In addition we believe that healthcare workers are typically more careful due to their knowledge of the disease and how it spreads. Access to protective gear is modeled with the parameter *ρ*.

**Table 1:** Variable and parameters common for all geographic locations.

Parameter, meaning	Value
*β*, basal transmission rates	optimized to fit data
Factors modifying transmission rate	
*ε*, asymptomatic transmission	0.75
*ρ*, reduced healthcare worker interactions	0.8
*ρ*_*v*_, reduced visitor-community interaction	0.5
*γ*, quarantine	0.2
*γ*_*v*_, quarantine for visitor	0.3
*κ*, hospital precautions	0.5
*η*, healthcare worker precautions	0.2375
Population fractions	
*p*_*i*_, *i* = 0,…,13, onset of symptoms after day *i*	0.000792, 0.00198, 0.1056, 0.198, 0.2376, 0.0858, 0.0528, 0.0462, 0.0396, 0.0264, 0.0198, 0.0198, 0.0198, 0
*q*_*s*,*i*_, *i* = 0,…,4, symptomatic isolation after day/stage *i*	C: 0.1, 0.4, 0.8, 0.9, 0.99; H: 0.2, 0.5, 0.9, 0.98, 0.99
r, transition to next symptomatic day/stage	0.2

In addition to standard SEIR parameters, we further parameterize the model to account for mitigation measures such as mask compliance and contact tracing, the latter being dependant on the geographical location. Table 2 lists the values we use for the State of Hawai‘i (those are assumed to be constant over the various counties) as well as the ones for three non-Hawaiian geo-locations included in our study. The parameters have been derived from dashboards/articles and, when possible, using contact tracing [19–28]. The choice of *q*_*a*,*i*_ reflects the various testing and contact tracing efforts, and gives the assumed probability for an asymptomatic individual to go into isolation as a result of testing and contact tracing.

**Table 2:** Geographically dependent factors modifying transmission rate.

Parameter, meaning	HI Counties	Japan	Puerto Rico	Iceland
Factors modifying transmission rate				
*p*_*mp*_, mask compliance	0.2 before Aug 27, 0.7 thereafter	0.2 before May 04, 0.8 thereafter	0.2 before Aug 21, 0.7 thereafter	0.2 before Oct 20, 0.5 thereafter
*p*_*me*_, mask efficiency	0.25	0.25	0.25	0.25
Population fractions				
*q*_*a*,*i*_, *i* = 0,…,13, asymptomatic isolation after day *i*	0 before Jun 08, then *q*_5_ = *q*_6_ = *q*_7_ = 0.05	0 before Feb 25, then *q*_5_ = *q*_6_ = *q*_7_ = 0.05	0 before May 05, then *q*_5_ = *q*_6_ = *q*_7_ = 0.05	0 before Apr 01, then *q*_5_ = *q*_6_ = *q*_7_ = 0.05

Simulations of our generalized SEIR model are done using our own implementation written in Python. An outline of the program structure is provided in S2 Appendix. The code is available upon request.

#### Initial conditions

The initial values of most variables are zero. The only non-zero initial values are the number of susceptible individuals in both the general community and the healthcare worker community compartments. These values are listed in Table 3. The count of healthcare workers in the State of Hawai‘i comes from [29] and some newspaper articles listing numbers to divide them per county. As an initial trigger, a single not isolated symptomatic individual, *I*_*c*,0_(0) = 1 is listed with the corresponding date of introduction into the model for each region.

**Table 3:** Susceptible population for each region and first detected symptomatic individual. All other variables have an initial value of 0. Kaua‘i is not represented in this table since the model cannot be implemented for this county due to the very low count of daily cases.

Region	*S*_*c*_(0)	*S*_*h*_(0)	Date for *I*_*c*,0_(0) = 1
Honolulu	937711	15000	Mar 06
Maui	167417	1500	Mar 15
Hawai‘i	201513	1500	Mar 16

Table 4 provides the initial values for Iceland, Japan and Puerto-Rico used in our modeling.

**Table 4:** Susceptible population for the three non-Hawaiian geo-locations.

Region	*S*_*c*_(0)	*S*_*h*_(0)	Date for *I*_*c*,0_(0) = 1
Japan	126500000	1673518	Feb 01
Iceland	356991	1404	Feb 21
Puerto Rico	3194000	89000	Mar 04

### Metrics for daily cases curves comparison

Critical to comparing the spread of the virus at different locations is the introduction of an appropriate comparison metric. We analyze similarity between the counties of Hawai‘i by computing the classical *L*_2_ norm for normalized model fits, with the time interval re-scaled to [0, 1]. Considering that we have a single sample for each of the time series representing the new daily cases, we assess the significance of the computed *L*_2_ distances by looking at how the corresponding numbers change when the model is fitted to random perturbations of the original time series data. These perturbations are obtained by adding Gaussian noise with mean zero. Assuming that a large number of new daily cases implies a larger possible error, the standard deviation for the noise is time dependent and equal to one-tenth of the recorded daily cases. In the rare cases when adding the noise yielded a negative number of new daily cases, this number is set to zero.

To assess how the shape of a curve changes from one time period to another, we look at the difference between the normalized *L*_2_ norms. Utilizing the generated permutations for all the counties, we can then perform a permutation test [30] and estimate a *p*-value for the hypothesis that the change is the same for all the counties. See [31] for a general discussion on distance measures to effectively determine similarity between trajectories, and [32–36] for applications to concrete problems using especially the *L*_2_ or more generally the Minkowski distance.

To quantify similarity between our counties and Japan, Iceland, Puerto Rico we use a slightly different metric. We also ran a fit with our compartmental model for these three other geographic locations and analyze similarity by looking at the qualitative structure of the results as captured by *merge trees* (see e.g. [37]). The latter construct is a topological descriptor of functions, and is constructed by tracking how connected components of the sublevel sets appear and merge as the threshold for the sublevel sets increases. This comparison is better represented with a standard *L*_2_ metric due to time shifts in the course of the pandemic for the various countries. An easy way to visualize this process is to move a horizontal line from the bottom to the top of the graph of a function and keep track of the function values at which a new connected component of the graph appears under the line or two existing components get merged. The actual horizontal locations of the branches, which represent the connected components, is not important. Rather their relative (left-right) positions are key. Unfortunately, assessing sensitivity of the computed merge trees to noise is significantly more challenging than in the case of the *L*_2_ distance. The main issue is that while one can define a metric on the space of merge trees, computation of this metric is a very complicated problem [37]. Nevertheless, we can try to informally assess the sensitivity by looking at how the definition of the distance between merge trees applies to the merge trees obtained for the model fitted to perturbed time series (as described above).

## Results

In this section we provide the results from the simulations of our model for the counties of the State of Hawai‘i under analysis. We display the raw daily cases and not the 7 day average because our model fit plots the sum over all groups of the newly isolated and isolated daily exposed as well as infected individuals. Individual model fits for each county as well as the corresponding optimized transmission rates can be found in S3 Appendix. For Kaua‘i county the daily cases are too small to generate a model, we therefore only use the raw daily cases.

**Fig 5:**
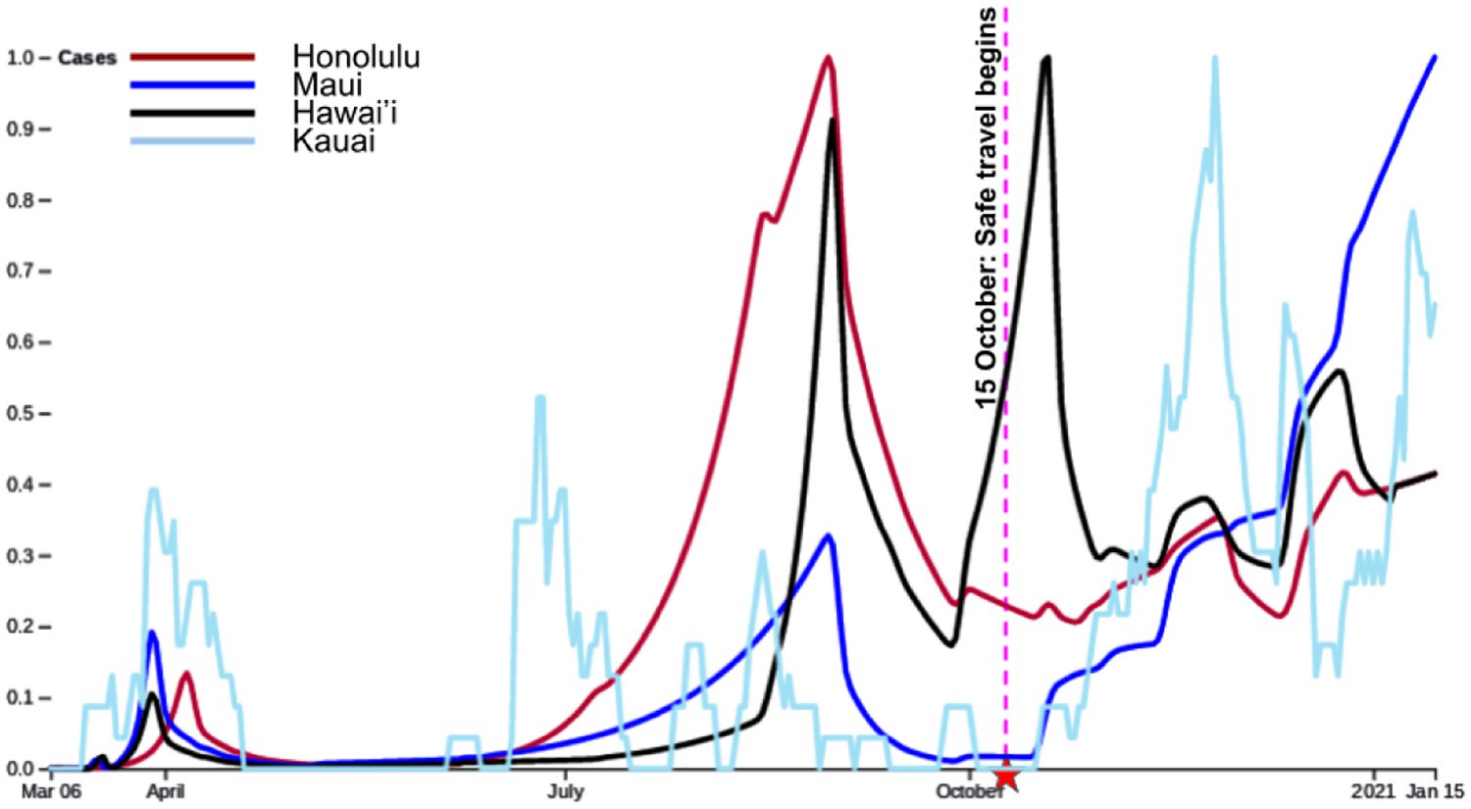
Honolulu, Maui and Hawai‘i counties with a normalized model fit, Kaua‘i with normalized daily cases. It is clearly observed that counties started to differ in response to the spread of COVID-19 after the Safe Travels Program opened.

As we hypothesized, there are clearly major differences among the four counties. Fig 5 shows, on the same plot, the normalized model fits for Honolulu, Hawai‘i and Maui counties as well as the daily raw numbers for Kaua‘i (each island is normalized individually by their max daily cases). It can be observed that (excluding Kaua‘i for which numbers have been too low to draw comparison) the spikes in case rates in the three main counties correlate well with each other until the Safe Travels Program began on October 15, 2020. Each of these counties have a spike preceding this date. Hawai‘i county displays the sharpest spike, which was attributed to a specific pair of clusters (one in the town Hilo and one in the residential area Ocean View). Maui also had a few clusters, including a major one around October 20 on Lanai and another major one in early January in the town of Kahului. After October 15, 2020, both Honolulu county and Hawai‘i county show a slight increase (Hawai‘i has a localized spike that initiated just before the safe-travels program and was identified to a known cluster). This is in contrast to Maui county, which displays a very sharp increase. The optimized transmission rates provided in S3 Appendix show that the exponential growths and decays for Hawai‘i and Maui counties require typically larger values for the basal transmission rate than for Honolulu county. We believe that the reason for this is that changes occur more rapidly in the outer-islands. For instance the peak for Honolulu county is based on a build-up starting in June, while for Hawai‘i county the peaks are much more narrower. For Maui county the decay due to the second stay-at-home order is extremely efficient at the beginning and then slows down. This forces an increase in *β* for an appropriate data fit.

**Fig 6:**
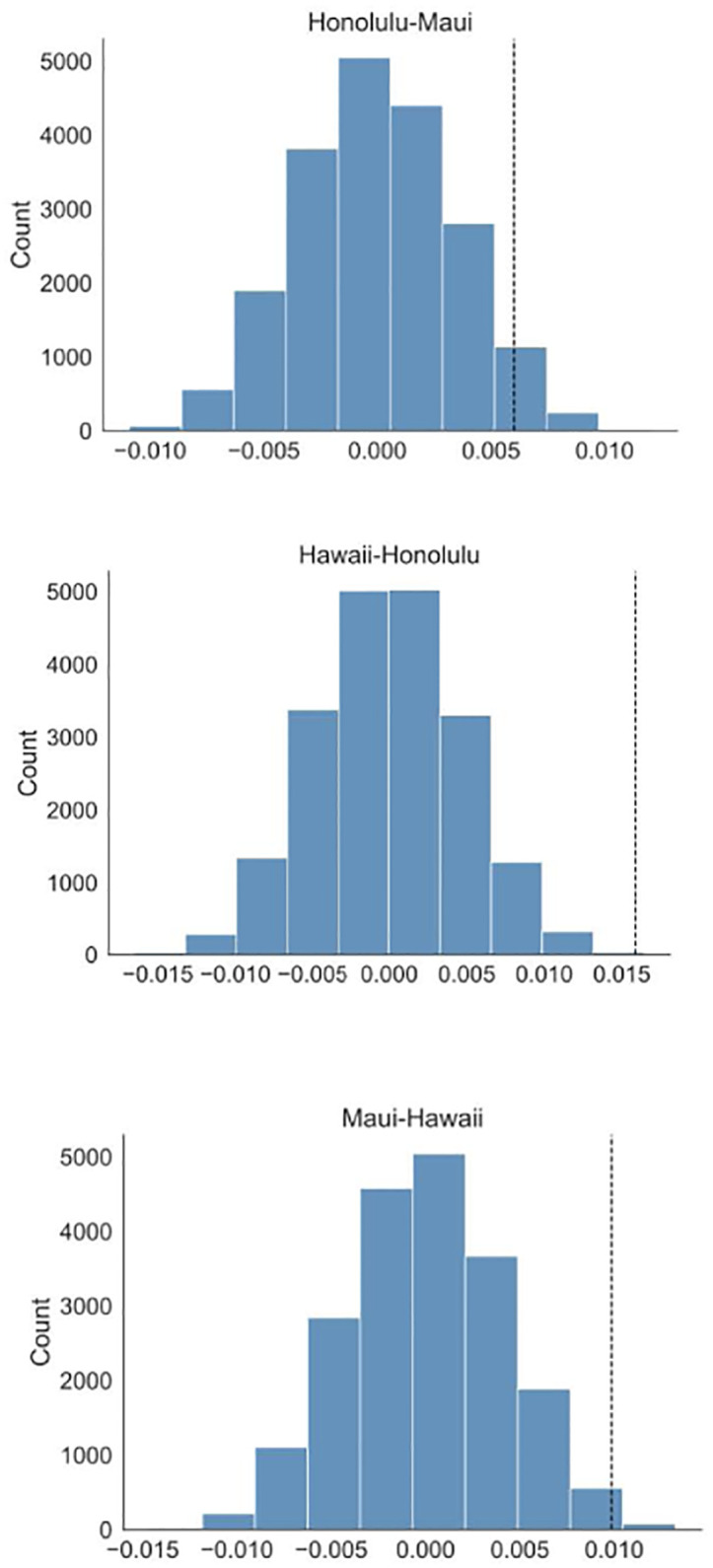
Estimated distribution of permuted difference, Δ, between the mean differences in *L*_2_-norms for the intervals before Oct. 15 and the one until Jan 15 for the three pairs of counties. The observed values, shown by black vertical lines, clearly suggest that the hypotheses of equality of the mean differences for the three pairs of counties should be rejected.

For each county, the perturbed normalized fit is considered over two time intervals: into two groups: before the safe travels (October 15) and until January 15, 2021. Then the mean difference between the *L*_2_ norms (with intervals re-scaled to [0, 1]) is computed for each county over all perturbations (see Table 5). For a pair of counties, we take the null hypothesis to be the equality of the means of these differences. We then perform a permutation test (with 20000 random permutations) to estimate the *p*-value for such a null hypothesis, as shown in Fig 6 and Table 6.

**Table 5:** Means for perturbed normalized *L*_2_ norms and their differences for Hawaiian counties computed over the time periods until Oct. 15 and until Jan 15.

	Mar 06–October 15	Mar 06–Jan 15	Difference
Honolulu	0.326	0.367	0.041
Hawai‘i	0.250	0.297	0.047
Maui	0.257	0.314	0.057

**Table 6:** The difference, Δ, between the mean differences in *L*_2_-norms for the intervals before Oct. 15 and the one until Jan 15, along with the *p*-value estimated using the permutation test.

	Δ	*p*-value
Honolulu—Maui	0.00601	0.0422
Hawai‘i—Honolulu	0.01588	0.0001
Maui—Hawai‘i	0.00987	0.00675

**Fig 7:**
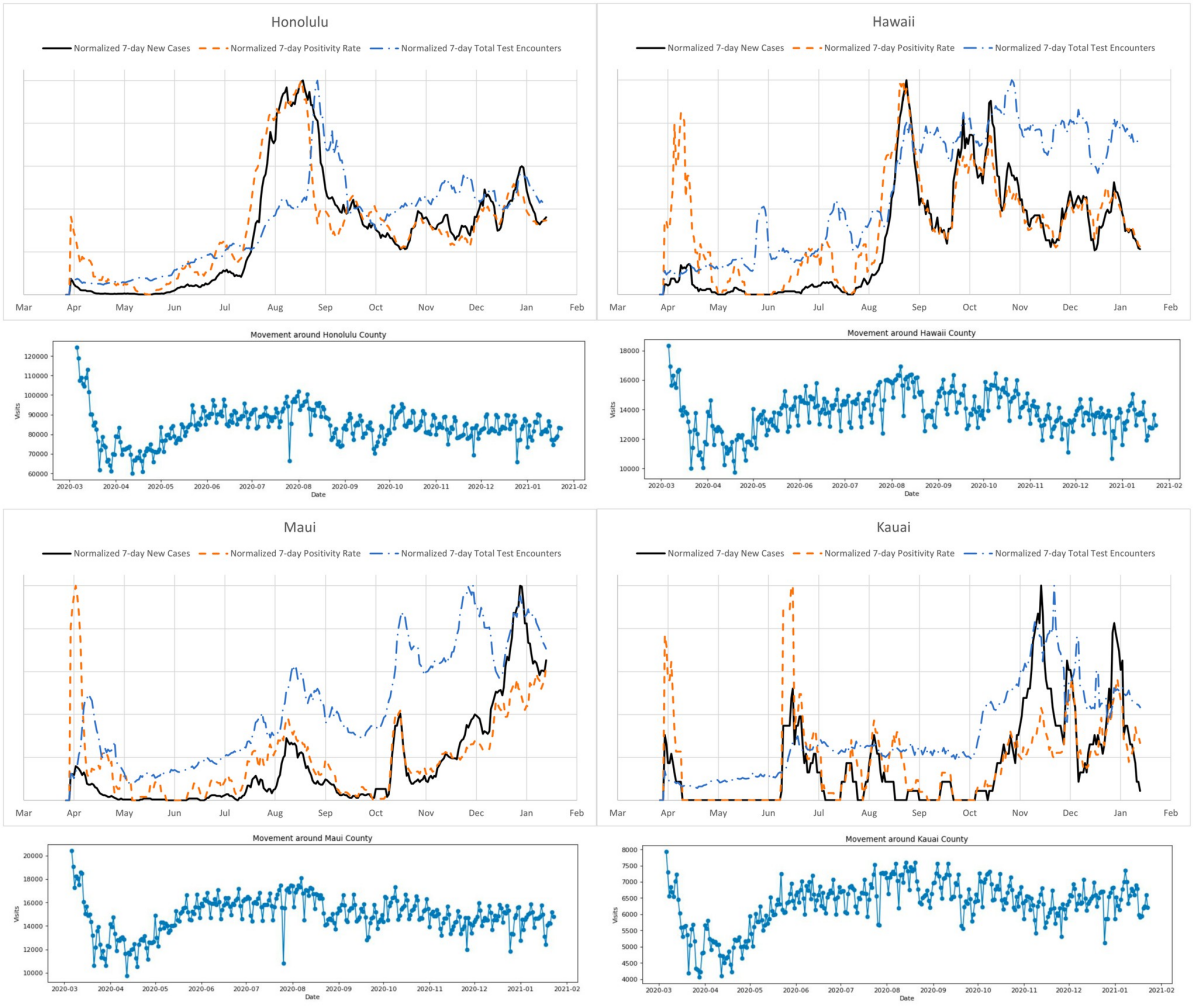
Testing, positivity and mobility plots. A: Honolulu. A sharp increase in the test positivity rate (along with the daily cases) in July indicates an outbreak of the disease. The later decrease in the positivity rate with the increased number of tests indicates a substantial slowdown of the spread of the disease. B: Hawaii. A sharp increase in the test positivity rate around August indicates an outbreak the disease. The later decrease in the positivity rate with the number of tests hovering around the same value indicates a welcome slowdown of the spread of the disease. Maui: A series of ups and downs in the test positivity rate and the number of daily cases indicate the occurrences of outbreaks of the disease. The significant increase in these numbers at the beginning of this year suggests a serious spread of the virus. A noticeable jump in the daily case number that does not correlate with the positivity rate can be explained by a jump in the number of tests, since the latter are performed for people with higher chances of having the virus. C: Kauai. The number of daily cases and test positivity rate are still well correlated, even though the raw numbers are small. Similar to Hawai‘i county, we can see a jump in the daily case numbers that correlates with the increased number of tests rather than the test positivity rate, which is likely due to the biased nature of the population sample on which the tests are performed. Overall mobility suggest a modest correlation with the number of daily cases. It shows a major dip in mobility triggered by the first stay-at-home order back in March 2020. The mobility data clearly suggests why the second lockdown was not as efficient as the first one.

**Fig 8:**
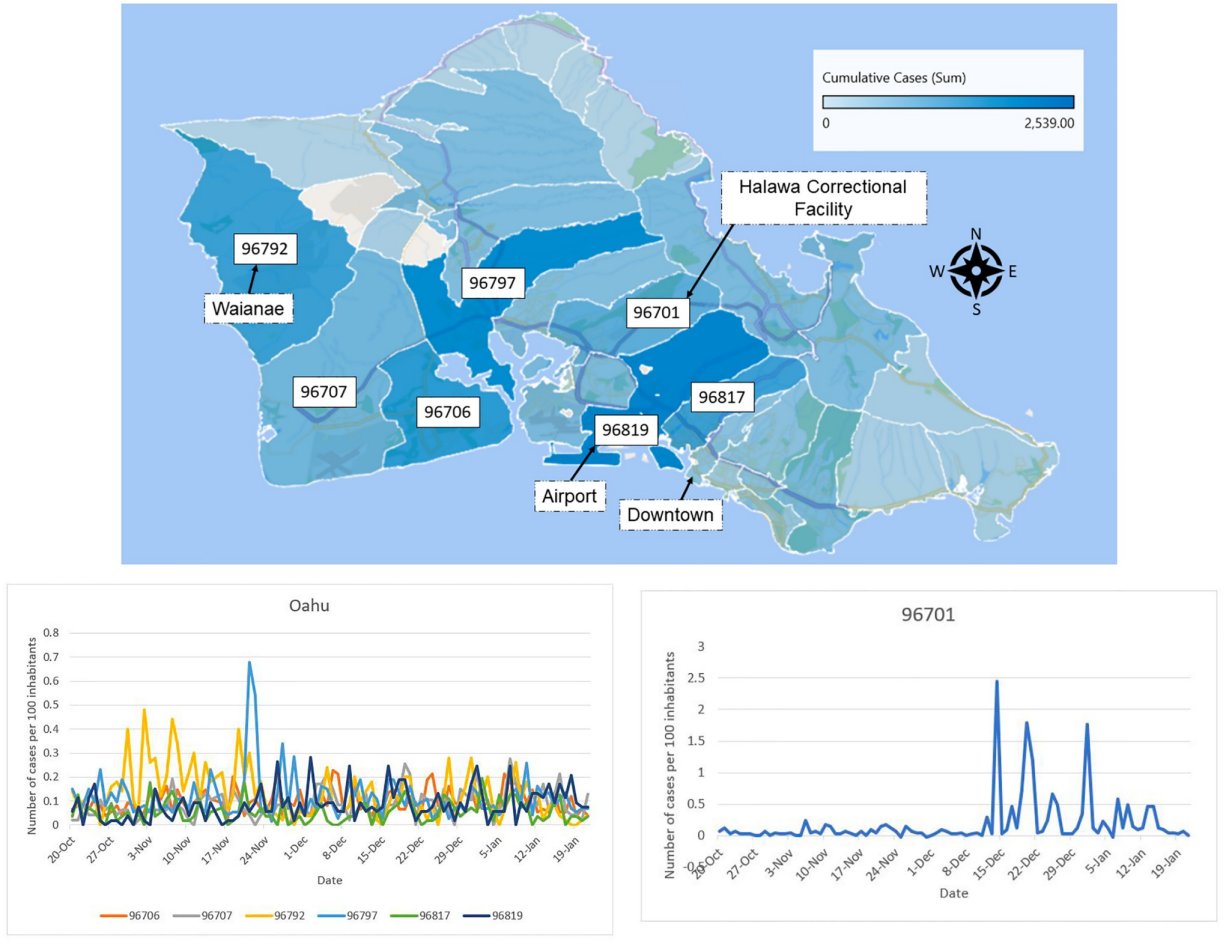
Honolulu county cumulative daily counts distributed per zip code from March 2020 to January 18, 2021. A: Map produced using Excel Map charts). B: Honolulu county cumulative daily counts distributed per zip code.

Fig 7 shows the total number of tests, the test positivity rate (i.e., the percent of tests for COVID-19 that came back positive) as well as the daily cases for each county following the description in material and methods. The noticeably large initial values of the test positivity rate (present for all counties) are likely caused by the a small number of test that have been administered to a very narrow slice of the population with much higher chances of having the virus. When interpreting these plots, it should also be noted that even later in the pandemic the sample of people receiving tests is not unbiased, since the State of Hawai‘i has been administering the test to people who satisfy criteria that make them more likely to have the virus. In general the movement between counties dramatically slowed down right after the onset of the pandemic. Afterwards, it rises slightly and then fluctuates with a movement index between the shutdown and almost normal rates. Curiously, the correlation between the mobility index and the daily case is far from strong, and in the case of Kaua‘i county the picture is more similar to anti-correlation. It suggests that the spread of the virus among households, especially large and multi-generational, could significantly contribute to the overall daily cases. Delving more deeply into the spread within counties, we use case rate data binned by zip codes. We find that for Honolulu county, Honolulu downtown as well as the West Coast (Waianae) have been the most affected in terms of daily cases. See Fig 8A. For the West Coast it is possibly due to its high pacific islanders population and the fact that they have been disproportionately impacted. While they form about 4% of the total Hawai‘i population they account for more than 27% of total cases [38]. Zip code 96819 dominates the count per 100 inhabitants, containing Moanalua, Kalihi, Kapalama, and Daniel K. Inouye International Airport on the south side of Oahu. A second area of interest is 96792 of the Waianae area on the west side of Oahu. From Fig 8B, we see that zip code 96701 displays a cluster behavior and that almost all its cases happened between December 16, 2020 to January 6, 2021. This was due to a cluster at Halawa Correctional Facility. There is no real immediate visible pattern from the other zip codes.

**Fig 9:**
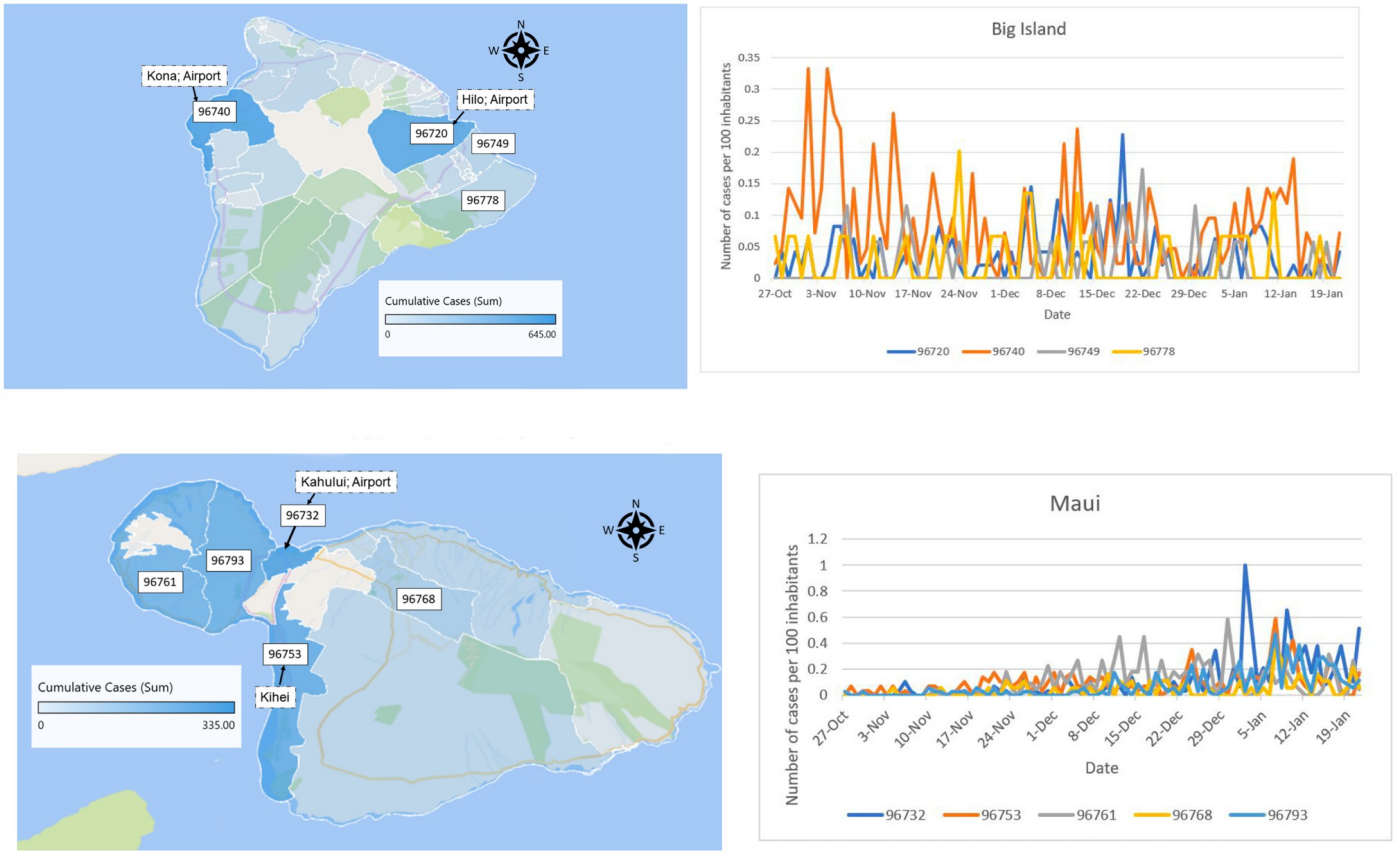
Hawai‘i and Maui cumulative daily counts distributed per zip code from October 2020 to January 18, 2021. A. Hawaii County. Cases restricted to the two major towns Hilo and Kona. B. Maui county. There were a few clusters on Maui which is an explanation for some of the higher spikes, in particular in early January in Kahului which is zip code 96732.

For Hawai‘i county the vast majority of cases are located in one of the two main town: Kona (West) and Hilo (East). Zip codes 96720 and 96740, respectively Hilo and Kona, clearly dominate the counts. We can see on Fig 9A that Kona had a consistently larger case rate than Hilo except for the few days before Christmas. This can might be explained by the fact that during the period October 15 to January 18, air traffic was more significant into Kona than into Hilo. The incoming air travel counts are as follows(tourist, returning resident): Kona (76189,23824), Hilo (15808, 8800). Each island also displays nonuniform population density and demographics. The low counts on the eastern half of Maui are likely associated with low population density of local residents and relatively few tourists Fig 9B. There was a large outbreak in a multistory building in early 2021 located in zip code 96732. (Multistory buildings with elevator usage are relatively rare on Maui.) This outbreak and spread has been attributed by authorities to elevator usage by the building residents in this complex, an uncommon phenomenon on Maui. There are a relatively larger number of tourists compared to local residents in zip codes 96761 and 96753 as compared to most other Maui zip code areas. Tourist travel is a possible reason these two zip code area had larger increases in December than other areas. See S3 Appendix for tables with the numbers for each counties.

**Fig 10:**
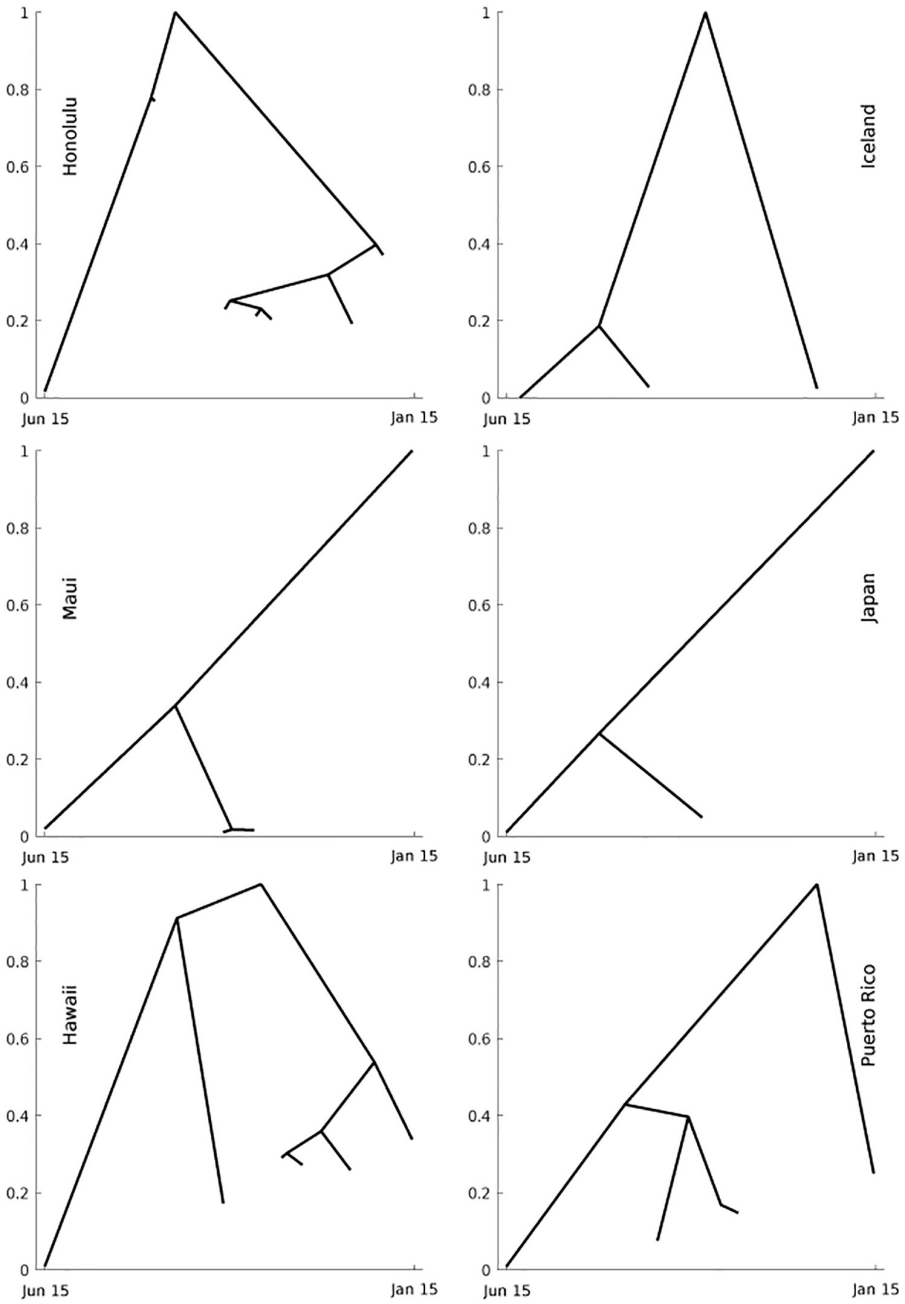
Merged trees. Merge trees elucidate the qualitative structure of the daily case numbers over time.

We now compare our counties to Japan, Iceland and Puerto Rico using the notion of merged trees. The merge trees for our Honolulu, Hawai‘i and Maui counties and the comparison geo-locations are shown in Fig 10. They are computed using the normalized time series for the daily cases numbers starting from June 15. We juxtaposed the merges trees based on their visual semblance and provide more details regarding their comparison in the Discussion section.

**Fig 11:**
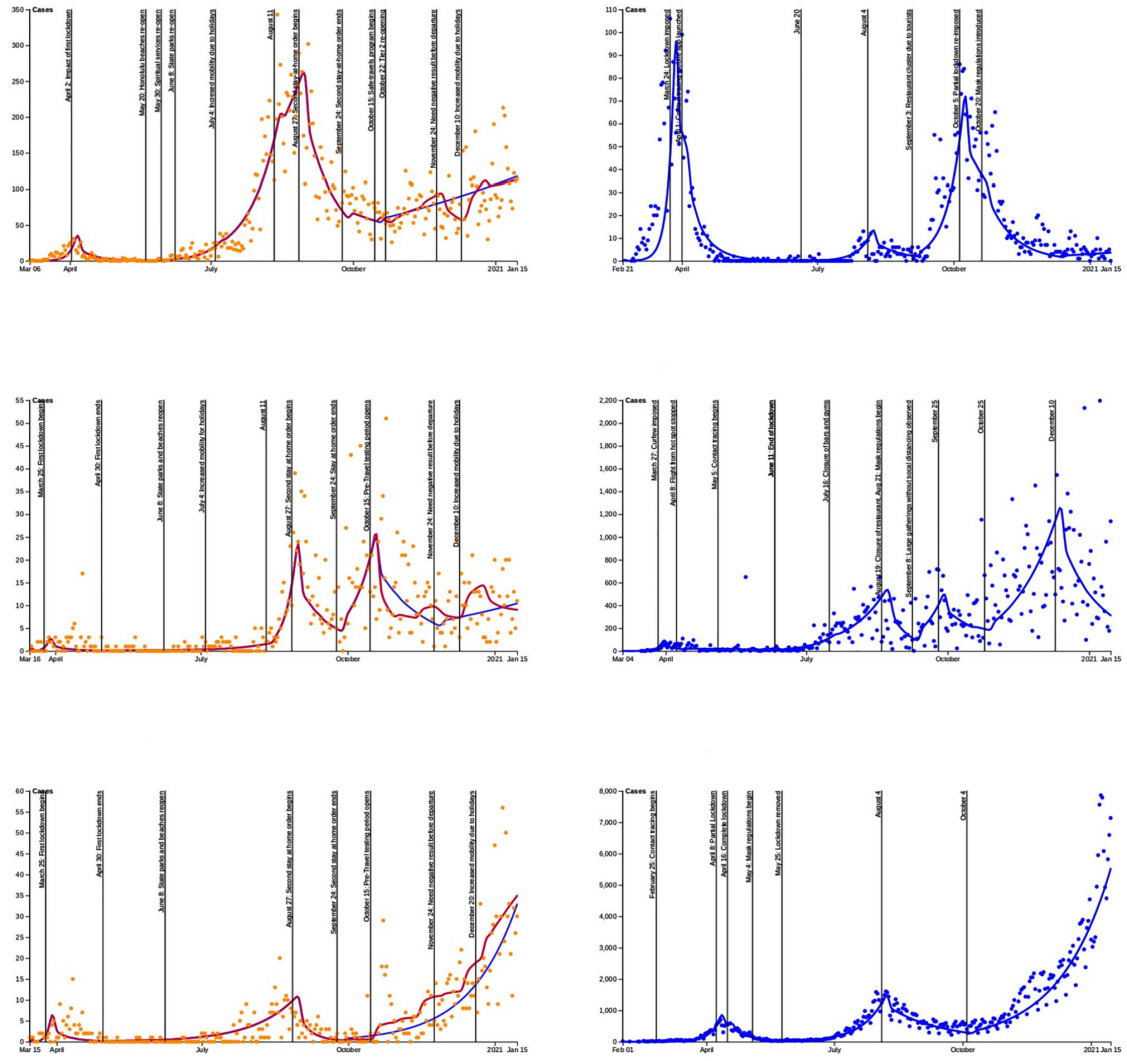
Comparison between the daily cases between Hawai‘i counties and other geo-regions. A: Honolulu County and Iceland. B. Hawai‘i County and Puerto-Rico. C: Maui county and Japan. Dots are daily cases and the curves are the computational fit using our compartmentalized model. The blue fit for the Hawai‘i counties starting October 15, 2020 corresponds to not taking into account the travelers.

Fig 11A–11C display the model fit for Honolulu, Hawai‘i and Maui counties along with the Islands paired to them using the merge trees of Fig 10. Iceland, most similar to Honolulu County, detected their first case in February and had a significant first wave, but then controlled the spread beside a super spreader event trigger by two travelers. Traveling has then be very restricted which is why the daily cases are mostly in the single digits at the end of the fit. Hawai‘i county is most similar to Puerto Rico. The accuracy of the data for Puerto Rico is unclear and it was very difficult to find the travel restrictions. The primary difference is the peak in December that Puerto Rico suffered. Maui and Japan display a very similar qualitative curve, especially when travelers are ignored for Maui. We believe the reason for Japan’s explosive growth at the end of the year is likely attributed to a few factors, including a controversial policy encouraging domestic travel and possibly COVID-19 mitigation fatigue by the population. We note that the travel policy was made more restrictive in January.

## Discussion

Understanding the impact of a pandemic on isolated locations is of extreme importance not only to the locations themselves but also can shed light on pandemic spread generally because of the lesser number of independent parameters. Due to their isolation, populations on islands or archipelagos are very vulnerable to pandemics and are potentially subject to devastating effects because of their isolation and often limited supply chains and hospitalization capacity. In this paper, using the Hawaiian archipelago, we explore the importance of taking into account local variations in island chains. In characterizing the individualistic island behavior, we believe that ratios between residents and tourists, age demographics and differences in governmental application of controls may account for the substantial noted differences.

The Hawaiian island chain has been significantly impacted by disease throughout their history. See, for example, the recent popular article in the Smithsonian Magazine which notes that, “Foreign diseases have come through here before, and they have inflicted unfathomable damage” [39]. COVID-19 is therefore a major threat for the State of Hawai‘i. While the majority of the population lives on Oahu, there are seven inhabited islands. The State numbers during the pandemic have been dominated by daily cases in Honolulu County, which overshadowed the spread of the disease in the other counties. Our results clearly illustrate that it is not sufficient to average the initial conditions of the virus spread and assume that the different islands will exhibit similar behavior in an average sense. On the contrary, nonlinear effects and clusters can take off in one of the contained populations at a different time, thus requiring different pandemic control mandates. This highlights the need for targeted measures at the county(island) level, especially once travel restrictions are started to be lifted. Indeed, during the initial stretch of the pandemic, there was very little inter-island travel due to a mandatory 14-day quarantine that was in place. (It was dropped in June 2020, but then reinstated in August 2020.) Travel picked-up again in October 15 with the Safe Travels Program in place. As illustrated by the numbers in Table 6 the counties started to become more dissimilar once travel restrictions were eased, which supported by the fact that the estimated *p*-values reject the null hypothesis of the same change for all the counties. It also demonstrates how localized clusters have a larger impact on the less populated islands. This is clearly demonstrated by the fact that as of January 2021, Maui county went into an alarming trend despite sharing the same mitigation measures and travel restrictions as Honolulu and Hawai‘i county. (Note that in Hawai‘i, the islands themselves correspond roughly to a single county. There are not county lines dividing contiguous state interiors as on the Mainland).

More precisely, we can see that the Honolulu county merge tree is visually similar to the Iceland merge tree and the Maui county merge tree is visually similar to the Japan merge tree. While the Hawai‘i county and Puerto Rico merge trees are less visually similar than the previous pairs, they both feature a tall right branch connecting to the absolute maximum as well as a tall right subtree of the main left branch, and are even more visually dissimilar from other merge trees than from each other. Looking more carefully at the definition of the interleaving distance between merge tress (see [37]), the reader can get a better understanding as to why these visual similarities are likely to be supported by the actual interleaving distances. We should also mention that the interleaving distance is bounded from above by the standard sup-norm of the difference between the corresponding time series. We observed that fitting the model to perturbed data does not change the resulting time series much, especially in terms of the sup-norm. Hence, we expect that the interleaving distances between the merges trees obtained for perturbed data are likely be very close to the distances between the merge trees shown in Fig 10. An example of merge trees computed for perturbed data is provided in S2 Fig. It shows that these merge trees are indeed very visually similar to the ones obtained for unperturbed data.

It is somewhat surprising that islands with control over the influx and outflow of people may have a similar overall pandemic experience even if they are geographically distant and culturally different. Merge trees indicate that the new daily cases curves for each of Honolulu, Hawai‘i and Maui counties display similar qualitative behavior to the corresponding curves for Iceland, Puerto Rico, and Japan, respectively. This suggests a much more complex dynamics for the spread of the disease within the island chain since similarity, climate, and proximity do not always correlate in terms of a disease spread behavior.

The State of Hawai‘i launched an aggressive mass vaccination campaign starting in December, but its effects are only now starting to impact the daily case rate. During the period of our study the very small impact of vaccination was ignored. As of February 2, 2021 we have 202,200 doses administered. The State policy is to keep the vaccination plan as originally planned, that is, administer two doses per individual, even though two cases of the more transmissible B1.1.7 have been detected in Hawai‘i. As of February 8, 2021, cases have been decreasing in all four counties. We note that the vaccination rate should also be adjusted to target specific counties in different ways (timeline, demographics, etc).

## Conclusions and future work

In this paper we present an in-depth study of the Covid-19 pandemic in the Hawaiian Islands. We pay special attention to details of the heterogeneity effects and compared individual island results with each other in Hawai‘i and with other islands outside of Hawai‘i to discern how much granularity and detail is appropriate for making policy decisions related to curtailing disease spread. We use the unique situation of the Islands’ officials ability to impose more effective quarantines and isolation to study the dynamics of the disease spread. We also use the paper to collect timely information that might otherwise be lost and directly related it to disease dynamics. This aids in connecting policy decisions aimed at curtailing viral spread with actual results in reduction of number of cases. Future work in this area should include more advanced modeling techniques that include better vaccine and tourist population dynamics as the pandemic progresses and hopefully wanes.

An important conclusion of this research is the identification of patterns that change extremely rapidly. This is due primarily to the nonlinear behaviour of the underlying equations that simulate the spread of the pandemic. We find that it is critical to assure that heterogeneity is included in modeling and thus decision making for adequate and effective pandemic control.

In addition to the daily cases, we looked at the cumulative daily counts for our Hawai‘i counties distributed per zip code from the onset of daily cases to January 18, 2021 (see Figs 8 and 9 and S3 Appendix). It shows that the cases are very localized. Not surprisingly, they are higher in urban locations and towns where the population density, as well as the probability of indoor gatherings, is higher.

Not studied in this paper is hospital bed capacity for the State of Hawai‘i. The county of Honolulu is home to most of the hospital facilities and healthcare workers. During the pandemic, COVID-19 patients with severe enough symptoms needed to be transferred to the hospitals on either Maui or Oahu. For example, after the outbreak on Lanai, patients with severe symptoms had to be transported to either Oahu’s or Maui’s hospitals. The primary reason we did not consider this subject here is the lack of consistent and clear data regarding hospitalisations. Similarly, quantification of COVID-19 related fatalities in the State of Hawai‘i is challenging and delicate. For instance, in January about 60 deaths have been reclassified and added to the cumulative count.

## Supporting information

S1 Fig Demographic data for the four counties. Age demographic and ethnicity distribution per county.
(PDF)

S2 Fig Merge trees for perturbed data. An example of merge trees for the islands from Fig 10 computed for the model fitted to perturbed data.
(PDF)

S1 Table The state’s general statistics by county. The table provides population and area densities statistics by county.
(PDF)

S2 Table Data for travelers. The table provides the estimates used in our model for travelers for the State of Hawai‘i and comparing countries since the beginning of the Safe travel program on October 15, 2020.
(PDF)

S1 Appendix Data sources for COVID cases. This file provides details about the various sources used to collect COVID-19 cases.
(PDF)

S2 Appendix Model dynamics. This file provides details about the compartmental model that has been used for this paper.
(PDF)

S3 Appendix Simulations. This file contains results of our simulations per counties. We display the model fits along with the actual data and provide the optimized values for the basal transmission rate.
(PDF)
