# Supplementary material for: COVID-19 heterogeneity in islands chain environment
Source: PLoS One. 2022 May 18;17(5):e0263866. doi: 10.1371/journal.pone.0263866 (PMC9116625; doi:10.1371/journal.pone.0263866)
Supplement: S1 Fig — Age demographic and ethnicity distribution per county. (PDF) [file pone.0263866.s001.pdf]

The figure below provides the age demographic distribution per county. Honolulu county has a larger percentage of individuals between 20 and 40 years old while Hawai'i county is more represented in the 55-80 years old age group. It can also be observed that the Honolulu county has a larger relative Asian population compared to the other counties and a smaller relative Native Hawaiian and Pacific Islander population.

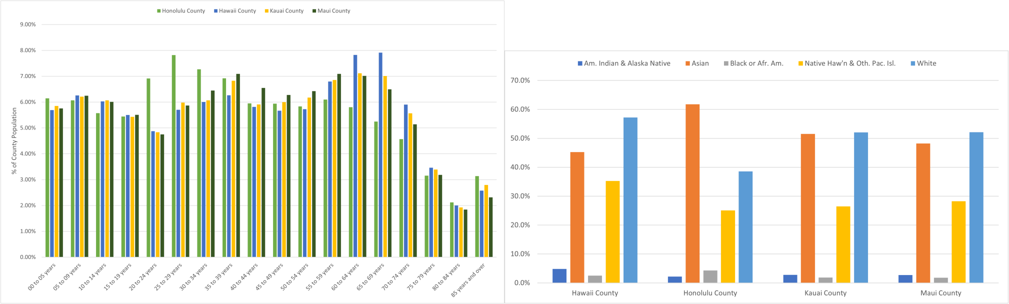

Left: Age demographic per county. Right: Ethnicity distribution per county. Hawai'i Data Collaborative, https://www.hawaiidata.org/covid19.
