# Supplementary material for: COVID-19 heterogeneity in islands chain environment
Source: PLoS One. 2022 May 18;17(5):e0263866. doi: 10.1371/journal.pone.0263866 (PMC9116625; doi:10.1371/journal.pone.0263866)

The figure below shows an example of merges trees obtained for our model fitted to perturbed data.

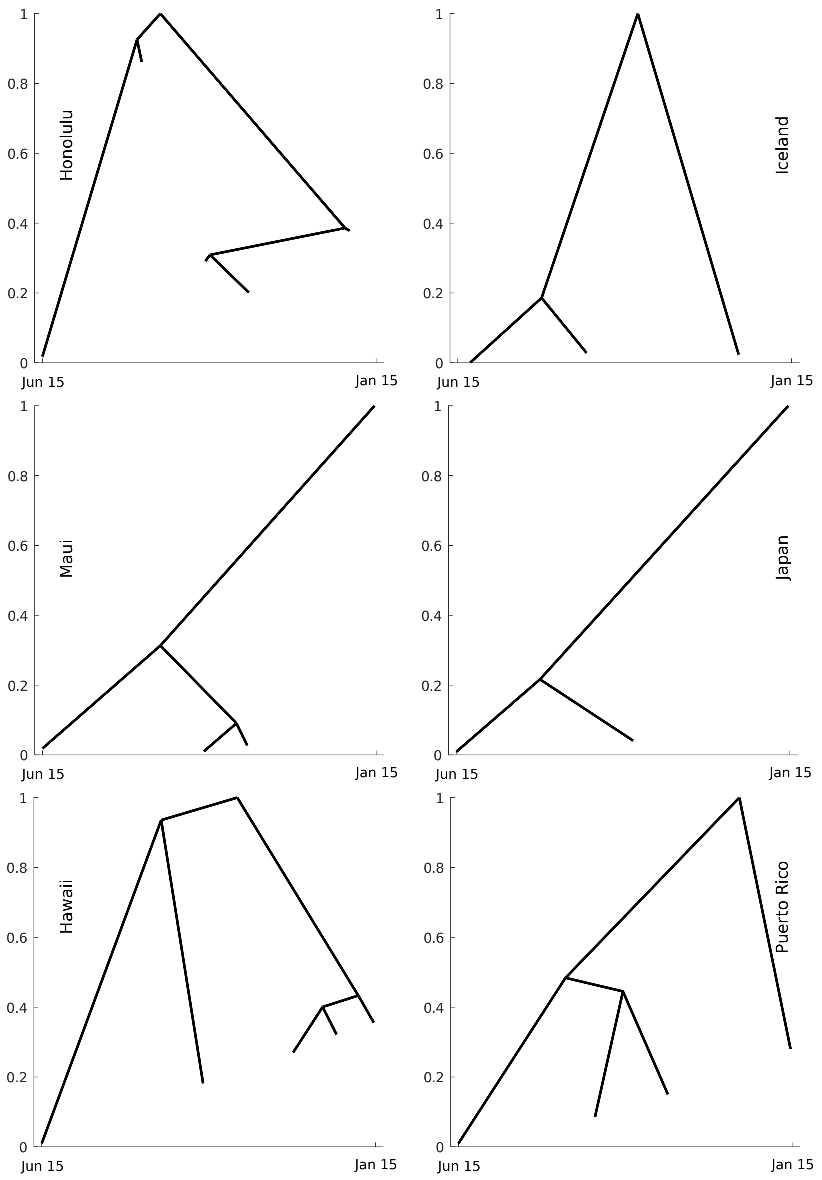

Supplement: S2 Fig — An example of merge trees for the islands from Fig 10 computed for the model fitted to perturbed data. (PDF) [file pone.0263866.s002.pdf]
