# Supplementary material for: COVID-19 heterogeneity in islands chain environment
Source: PLoS One. 2022 May 18;17(5):e0263866. doi: 10.1371/journal.pone.0263866 (PMC9116625; doi:10.1371/journal.pone.0263866)
Supplement: S1 Table — The table provides population and area densities statistics by county. (PDF) [file pone.0263866.s003.pdf]

Statistics (2019)	Honolulu	Hawai‘i	Maui	Kaua‘i	Source
Land Area (square miles)	600.74	4,028.42	1,161.52	619.96	DBEDT [2]
Resident Population	974,563	201,513	167,503	72,293	DBEDT [1]
Resident Population State Percent	68.8%	14.2%	11.8%	5.1%	Calculated
Tourists (thousands per year)	5862.4	1706.2	2914.9	1388.6	DBEDT [1]
Tourists (as daily percent of residents)	1.64%	2.32%	4.77%	5.26%	Calculated

The state's general statistics by county.

References

1. Economic Data Warehouse. Research & Economic Analysis | Economic Data Warehouse. State of Hawai‘i (2021). https://dbedt.hawaii.gov/economic/datawarehouse
2. The State of Hawaii Data Book 2014. Economic Data Warehouse State of Hawai‘i (2014). https://files.hawaii.gov/dbedt/economic/databook/2014-individual/01/011114.pdf
