# Supplementary material for: COVID-19 heterogeneity in islands chain environment
Source: PLoS One. 2022 May 18;17(5):e0263866. doi: 10.1371/journal.pone.0263866 (PMC9116625; doi:10.1371/journal.pone.0263866)
Supplement: S2 Table — The table provides the estimates used in our model for travelers for the State of Hawai‘i and comparing countries since the beginning of the Safe travel program on October 15, 2020. (PDF) [file pone.0263866.s004.pdf]

The safe travel program started on October 15, 2020 which is when travelers are implemented in the model (they were negligible before that). See Table **??** for the values used on our model.

Region	Tourists	Returning Residents	Health Workers
Honolulu	[1353,2124,3051,2028,4724,2195]	[692,716,967,951,1014,1018]	14500
Maui	[800,1000,2000,1700,3000,2500]	[128,127,135,158,160,156]	1500
Hawai'i	[297,593,981,751,1712,1000]	[116,113,108,136,124,128]	1500
Japan	700	700	
Iceland	0	0	
Puerto Rico	3500	500	

Average visitors per day, starting on October 15, 2020 to January 15, 2021.

References

1. KHON2. First wave of cuts hits state-owned hospitals in East Hawaii. (2015). https://www.khon2.com/local-news/first-wave-of-cuts-hits-state-owned-hospitals-in-east-hawaii/amp/

2. Kona Community Hospital. Careers. (2021). https://kch.hhsc.org/careers/

3. Star Advertiser. Cluster of workers at Maui Memorial Medical Center have contracted coronavirus, Mayor Victorino confirms. (2020). https://www.staradvertiser.com/2020/04/08/breaking-news/cluster-of-workers-at-maui-memorial-medical-center-have-contracted-coronavirus-sources-say/

4. U.S. Bureau of Labor Statistics. Hawaii - May 2020 State Occupational Employment and Wage Estimates. (2020) https://www.bls.gov/oes/current/oes_hi.htm#29-0000
