# Supplementary material for: COVID-19 heterogeneity in islands chain environment
Source: PLoS One. 2022 May 18;17(5):e0263866. doi: 10.1371/journal.pone.0263866 (PMC9116625; doi:10.1371/journal.pone.0263866)
Supplement: S1 Appendix — This file provides details about the various sources used to collect COVID-19 cases. (PDF) [file pone.0263866.s005.pdf]

There are useful collections of Hawaiʻi COVID-19 data in the form of dashboards: the Hawaiʻi Emergency Management Agency's (HiEMA) dashboard, the State of Hawaiʻi 's Department of Health's Disease Outbreak Control Division's (DOCD) COVID-19 dashboard, and COVID Pau dashboard (CPD) [1–3]. Directly utilizing these dashboards alone is challenging. Firstly, the dashboards are not synchronized; they often display different data at various times for the same quantities, such as hospitalization data. Secondly, the availability of the dashboard data is sometimes restricted because of political concerns. Both HiEMA and DOCD provide visual data in plots, but do not allow for downloading of the data. The Hawaiʻi Data Collaborative dashboard [4] resolves a majority of these issues by providing a Google Spreadsheet of the local Department of Health's DOCD data. The Hawaiʻi Data Collaborative also works to coalesce data from the other dashboards, and even obtains data directly from the office of Lt. Governor Josh Green. Collected data and their sources are summarized in Table 1.

Statistic	Source
Daily Cases	Hawaiʻi Data Collaborative [4]
Deaths	Hawaiʻi Data Collaborative [4]
Testing Data	Hawaiʻi State Department of Health [2]
Hospitalization	Hawaiʻi Data Collaborative [4]
Infections by County	Hawaiʻi State Department of Health [2]
Mobility Index	Hawaiʻi State Department of Health [2]
Traveler Data	Hawaiʻi Data Collaborative [4]

Table 1. The sources of COVID-19 statistics for this paper. The Hawaiʻi State Department of Health data is original, while the Hawaiʻi Data Collaborative takes a large portion of it's data from the Department of Health.

In this paper, we also compare the Hawaiian counties to other countries, Table 2 summarizes the sources for the data we used.

Statistic	Source
Daily Cases Iceland	COVID-19 in Iceland - Statistics [5]
Daily Cases Japan	Japan COVID-19 Coronavirus Tracker [6]
Daily Cases Puerto-Rico	The COVID Tracking Project [7]

Table 2. The sources of COVID-19 data used in this paper for the comparison countries.

References

1. COVID-19 Information Hub. HiEMA COVID-19 Information Hub. State of Hawaiʻi (2020). https://hiema-hub.hawaii.gov/

2. Hawaiʻi COVID-19 Data. Disease Outbreak Control Division | COVID-19 | Hawaiʻi COVID-19 Data. State of Hawaiʻi (2021). https://health.hawaii.gov/coronavirusdisease2019/what-you-should-know/current-situation-in-hawaii/#summary-metrics

3. Data Creates Knowledge. Our Data - COVID Pau: Turning Data into Knowledge. Hawaiʻi Data Collaborative (2021). https://covidpau.org/our-data/

4. COVID Data Resources. Hawaiʻi COVID-19 Data Resources - Hawaiʻi Data Collaborative. Hawaiʻi Data Collaborative (2021). https://www.hawaiidata.org/covid19

5. COVID in Iceland. COVID-19 in Iceland Statistics. (2020) https://www.covid.is/data

6. Japan COVID-19 Coronavirus Tracker. COVID19 Japan Patient Database (2020). https://covid19japan.com/#confirmed
7. The COVID tracking project. The Data - Puerto-Rico. (2020) https://covidtracking.com/data/state/puerto-rico
