# Supplementary material for: COVID-19 heterogeneity in islands chain environment
Source: PLoS One. 2022 May 18;17(5):e0263866. doi: 10.1371/journal.pone.0263866 (PMC9116625; doi:10.1371/journal.pone.0263866)
Supplement: S2 Appendix — This file provides details about the compartmental model that has been used for this paper. (PDF) [file pone.0263866.s006.pdf]

The equations for the dynamics of the three population groups are essentially the same and are given below. Only the hazard rate and the parameters determining transition rates into quarantine may be different between the three groups.

$$S(t+1) = e^{-\lambda(t)} S(t) \tag{1}$$

$$E_0(t+1) = (1 - e^{-\lambda(t)}) S(t) \tag{2}$$

$$E_i(t+1) = (1 - p_{i-1})(1 - q_{a,i-1}) E_{i-1}(t), \quad i = 1, \ldots, 13 \tag{3}$$

$$E_{q,i}(t+1) = (1 - p_{i-1})(q_{a,i-1} E_{i-1}(t) + E_{q,i-1}(t)), \quad i = 1, \ldots, 13 \tag{4}$$

$$I_0(t+1) = \sum_{i=0}^{13} p_i (1 - q_{a,i}) E_i(t) \tag{5}$$

$$I_1(t+1) = (1 - q_{s,0}) I_0(t) \tag{6}$$

$$I_2(t+1) = (1 - q_{s,1}) I_1(t) + (1 - r)(1 - q_{s,2}) I_2(t) \tag{7}$$

$$I_j(t+1) = r(1 - q_{s,j-1}) I_{j-1}(t) + (1 - r)(1 - q_{s,j}) I_j(t), \quad j = 3, 4 \tag{8}$$

$$I_{q,0}(t+1) = \sum_{i=0}^{13} p_i (q_{a,i} E_i(t) + E_{q,i}(t)) \tag{9}$$

$$I_{q,1}(t+1) = I_{q,0}(t) + q_{s,0} I_0(t) \tag{10}$$

$$I_{q,2}(t+1) = I_{q,1}(t) + q_{s,1} I_1(t) + (1 - r)(q_{s,2} I_2(t) + I_{q,2}(t)) \tag{11}$$

$$I_{q,j}(t+1) = r(q_{s,j-1} I_{j-1}(t) + I_{q,j-1}(t)) + (1 - r)(q_{s,j} I_j(t) + I_{q,j}(t)), \quad j = 3, 4 \tag{12}$$

$$R(t+1) = R(t) + r I_4(t) + r I_{q,4}(t) + (1 - p_{13}) E_{13}(t) + (1 - p_{13}) E_{q,13}(t) \tag{13}$$

Below is a detailed description of the variables, all of which depend on time, t, measured in days.

- **Variable $S(t)$.** The number of susceptible individuals.
- **Variables $E_i(t)$.** The number of asymptomatic infected individuals i days after exposure who are not quarantined.
- **Variables $E_{q,i}(t)$.** The number of quarantined asymptomatic infected individuals i days after exposure.
- **Variables $I_j(t)$, $i = 0, 1$.** The number of symptomatic infected individuals i days after the onset of symptoms who are not quarantined.
- **Variables $I_j(t)$, $j = 3, 4, 5$.** The number of symptomatic infected individuals at the nominal stage i of the illness. Note that a person can stay at a given stage for several days.
- **Variables $I_{q,j}(t)$, $j = 0, 1$.** The number of quarantined symptomatic infected individuals, with j representing either the number of days after the onset of the symptoms ($j = 0, 1$), or the stage of the illness ($j = 2, 3, 4$).

- **Variable** $R(t)$**.** The number of removed (recovered or deceased) individuals.

Splitting exposed individuals into multiple stages, E_i, allows us to capture possible differences in the progression of the asymptomatic phase of the disease. Importantly, it allows us to take into account that, according to the Centers for Disease Control and Prevention (CDC) as well as other sources, about 40% of people who contract SARS-CoV-2 remain asymptomatic, and the incubation period for those who do develop symptoms is somewhere between 2 to 14 days after exposure, with the mean incubation period between 4 and 6 days [1–3]. Individuals who do not develop symptoms after 14 days are assumed recovered. The use of the quarantine sub-compartments, $E_{q,i}$, allows us to capture the effect of contact tracing and the reduced transmission rate for quarantined individuals.

Similarly, having multiple stages for infected individuals better reflects progression of the symptomatic phase of the disease. The first two stages represent the first two days of symptoms, but the next three should be understood as phases of the immune system fighting the disease. There is a substantial variability (due to age as well as other factors) in the number of days any given person can spend at each stage. Our model implicitly assumes that the symptomatic phase of the illness lasts at least 5 days (in the unlikely case that each stage lasts just one day).

As we mentioned, a crucial part of the dynamics relates to the hazard rate. For the general community, group C, we have

$$\begin{aligned}\lambda_c(t) = \beta(1-p_{mp}(1-p_{me}))\Big[&(I_c+\varepsilon E_c)+\gamma((1-\nu)I_{c,q}+\varepsilon E_{c,q})+\\ &\rho[(I_h+\varepsilon E_h)+\gamma((1-\nu)I_{h,q}+\varepsilon E_{h,q})]+\\ &\rho_v[(I_v+\varepsilon E_v)+\gamma((1-\nu)I_{v,q}+\varepsilon E_{v,q})]\Big]/(N_c+\rho_v N_v), \end{aligned} \tag{14}$$

and for the tourists we have

$$\lambda_v(t) = \frac{\rho_v\beta\lambda_c+\beta_v(1-p_{mp}(1-p_{me}))\Big[(I_v+\varepsilon E_v)+\gamma((1-\nu)I_{v,q}+\varepsilon E_{v,q})\Big]}{(\rho_v N_c+N_v)}, \tag{15}$$

where we suppressed the dependency on t on the right for convenience. We use sub-indices c (community), h (healthcare workers), and v (tourists) to indicate the appropriate group. Subscript q indicates quarantined individuals. Here p_{me} and p_{mp} represent mask efficiency and mask compliance. Mask efficiency is chosen to reflect a reduction in transmission of 75% for all regions. Mask compliance is set at 20% for all regions at the start of the pandemic, but this value is modified on the dates the regions introduce mask regulations. N_v denotes the mixing pool for the visitors and N_c denotes the mixing pool for the general community, computed as

$$N_c(t) = S_c+E_c+I_c+R_c+\rho(S_h+E_h+I_h+R_h)+\rho_{v1}(S_v+E_v+I_v+R_v). \tag{16}$$

where variables E and I here represent the sum over all the stages within these compartments. For the healthcare worker group, we have

$$\lambda_h(t) = \rho\lambda_c+\beta\eta\Big[(I_h+\varepsilon E_h)+\kappa\nu(I_{h,q}+I_{c,q}+I_{v,q})\Big]/N_h, \tag{17}$$

where $N_h(t) = S_h+E_h+I_h+R_h$.

The model fit plot the following value

$$\sum_{x=c,h,v}\Big(\sum_{i=1}^{3} q^x_{s,i}I(i)+(1-r)q^x_{s,4}I(4)+\sum_{i=1}^{1}2q^x_{a,i}E(i)\Big) \tag{18}$$

The diagram below illustrates the structure of our code for our model.

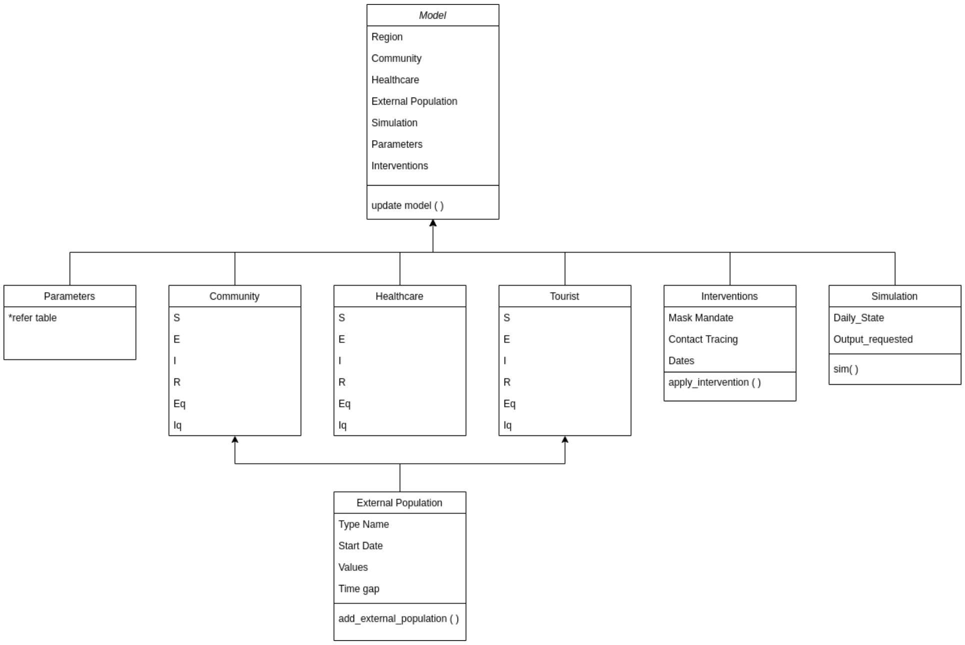

Illustration of the implementation of our code.

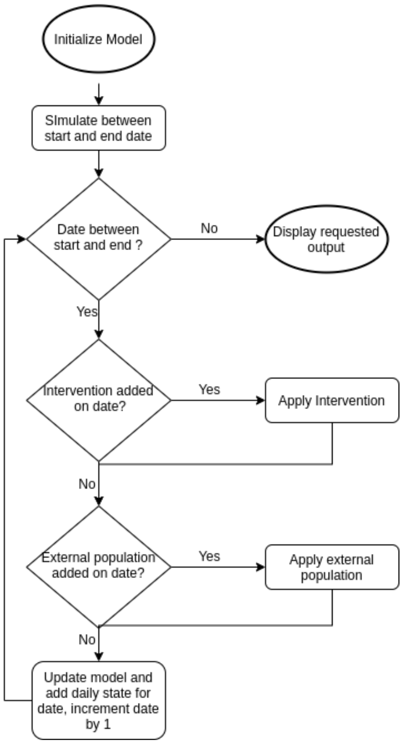

References

1. Moghadas S.M. et al. The implications of silent transmission for the control of covid-19 outbreaks Proc. Natl. Acad. Sci., vol. 117, no. 30, pp. 17 513–17 515, 2020

2. Oran D.P., Topol E.J. Prevalence of asymptomatic SARS-CoV-2 infection Ann. Intern. Med., vol. 173, no. 5, pp. 362–367, 2020

3. Park M., Cook A.R., Lim J.T., Sun Y., Dickens B.L. A systematic review of COVID-19 epidemiology based on current evidence. J. Clin. Med. 2020, 9 (4).
