# Supplementary material for: COVID-19 heterogeneity in islands chain environment
Source: PLoS One. 2022 May 18;17(5):e0263866. doi: 10.1371/journal.pone.0263866 (PMC9116625; doi:10.1371/journal.pone.0263866)
Supplement: S3 Appendix — This file contains results of our simulations per counties. We display the model fits along with the actual data and provide the optimized values for the basal transmission rate. (PDF) [file pone.0263866.s007.pdf]

Honolulu County

Figure 1 displays the model fit for the Honolulu county. The dots represents the daily cases and the curve is the model fit. The vertical lines corresponds to mitigation measures that had an impact on the curve and for which we optimized the β. Table 1 explicit the different β's. The maximal daily case for Honolulu county was 342 and happened on August 12, 2020. We see two major exponential growths, one early in March that was crushed through a stay-at-home order and one in August followed by a second stay-at-home order. However the second lockdown was lifted before daily cases reached single digits in the hope to save the local economy. It can be seen on Table 1 that the first lockdown was more efficient. The largest peak is attributed to the July 4 festivities, the transmission rate β was however quite smaller than for the first peak, but the State was much slower to call for a second stay-at-home order which resulted in the significantly higher counts. On October 15, 2020 the state of Hawai'i introduced the safe travel program which prompted an influx of tourists and traveling residents, this influx varies with time which explains the waving shape of the fit. Since the Safe travel program the daily cases have been fluctuating quite a lot which makes a fit difficult (some high daily cases came from a correctional facility cluster for instance).

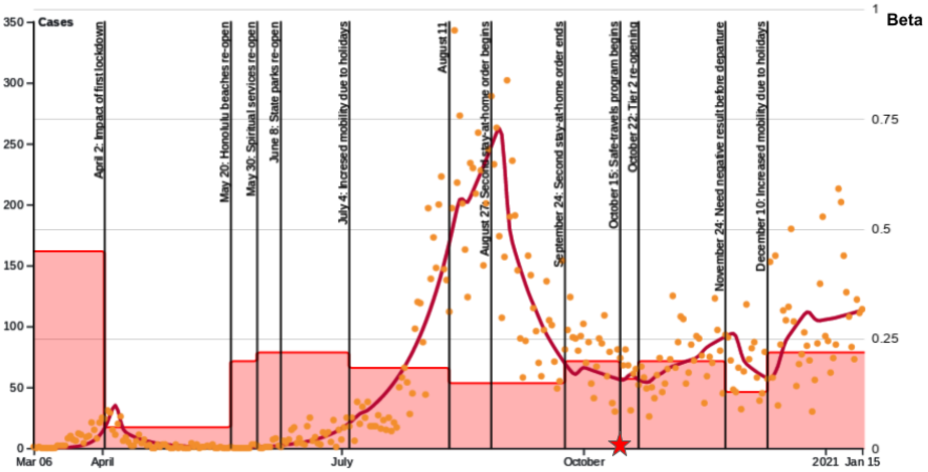

Fig 1. City and county of Honolulu: COVID-19 daily count and key events reflecting a change in behavior. The star shows the beginning of safe travel program.

Transmission rates			
March 6 - April 1	April 2 - May 19	May 20 - May 29	May 30 - Jul 3
$\beta = 0.45$	$\beta = 0.05$	$\beta = 0.2$	$\beta = 0.22$
Jul 4 - Aug 10	Aug 11 - Sep 23	Sep 24 - Oct 14	Oct 15 - Oct 21
$\beta = 0.185$	$\beta = 0.15$	$\beta = 0.2$	$\beta = 0.16$
Oct 22 - Nov 23	Nov 24 - Dec 9	Dec 10 - Jan 15	
$\beta = 0.20$	$\beta = 0.13$	$\beta = 0.22$	

Table 1. Optimized transmission rates to fit Honolulu county data. They reflect the State and Honolulu non-pharmaceutical mitigation measures.

Table 2 displays the largest zip code counts for Honolulu County.

Honolulu County Zip code	Population Estimate	Cumulative Daily cases	Cum. Daily cases per 100 inhabitants
96701	40857	1156	28
96706	74592	1562	21
96707	46928	850	18
96792	49971	1534	31
96797	73579	2038	28
96817	56144	1493	26.5
96819	52981	2342	44

Table 2. The seven zip codes with the largest cumulative distribution of daily cases.

Hawai'i County

Daily cases for Hawai'i county were very small until the aftermath of the July 4 celebrations which generated a large spike. The second stay-at-home order on Hawai'i was extremely efficient but immediately followed by an exponential increase in the form of a few clusters. The maximum value is 51 and happened on October 25, 2020 during the third peak with a very close value during the second peak of 39 on August 29, 2020. One can observe a somewhat puzzling decrease in the number of daily cases after the start of the safe travel program. A potential explanation is that the spike in the number of cases that happened at that time was an isolated event unrelated to other activities on the island.

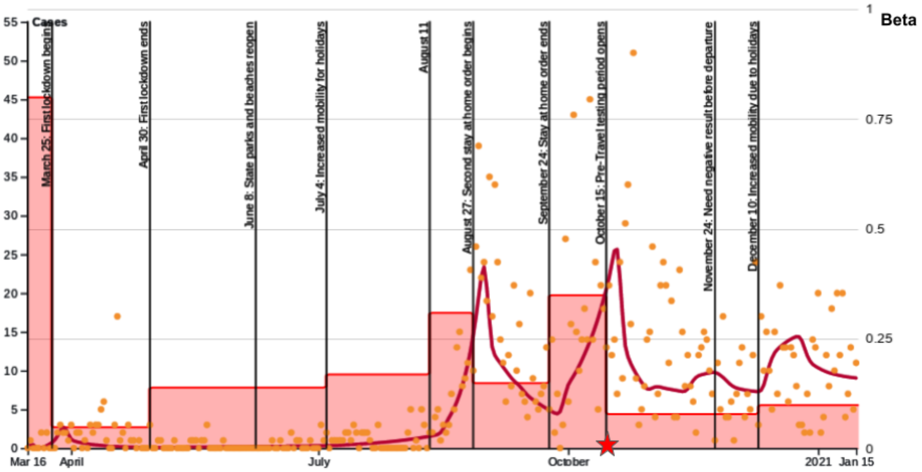

Fig 2. Hawai'i county: COVID-19 daily count and key events reflecting a change in behavior. The star shows the beginning of the safe travel program.

Table 4 displays the largest zip code counts for Honolulu County.

Maui County

Maui county started the pandemic with a relatively low number of daily cases, but then entered an alarming state of a high number of cases per hundred thousand of population even reaching a a maximum of 56 cases on January 6, 2021. It can be seen clearly the trigger with the introduction of the safe travel program on October 15, 2020. The influx of travelers is not constant through time and because the ratio tourists versus residents is high on Maui we see

Transmission rates		
Mar 16 - Mar 24	Mar 25 - Apr 29	Apr 30 - Jul 3
$\beta = 0.80$	$\beta = 0.05$	$\beta = 0.14$
Jul 4 - Aug 10	Aug 11 - Aug 26	Aug 27 - Sep 23
$\beta = 0.17$	$\beta = 0.31$	$\beta = 0.15$
Sep 24 - Oct 14	Oct 15 - Dec 09	Dec 10 - Jan 15
$\beta = 0.35$	$\beta = 0.08$	$\beta = 0.10$

Table 3. Optimized transmission rates to fit Hawaiʻi county data. They reflect the State and Hawaiʻi non-pharmaceutical mitigation measures.

Hawaiʻi County Zip code	Population Estimate	Cumulative Daily cases	Cum. Daily cases per 100 inhabitants
96720	48339	594	12
96740	42069	615	15
96749	17308	122	7
96778	14885	100	7

Table 4. The four zip codes with the largest cumulative distribution of daily cases.

as a result the wavy increasing curve. In addition to the effect of additional tourists there was a large outbreak in relatively high population density condominium complex. The initial increase after October 15 was solely due to travelers which is why we see a rise in daily cases even though the basal transmission rate β stays small.

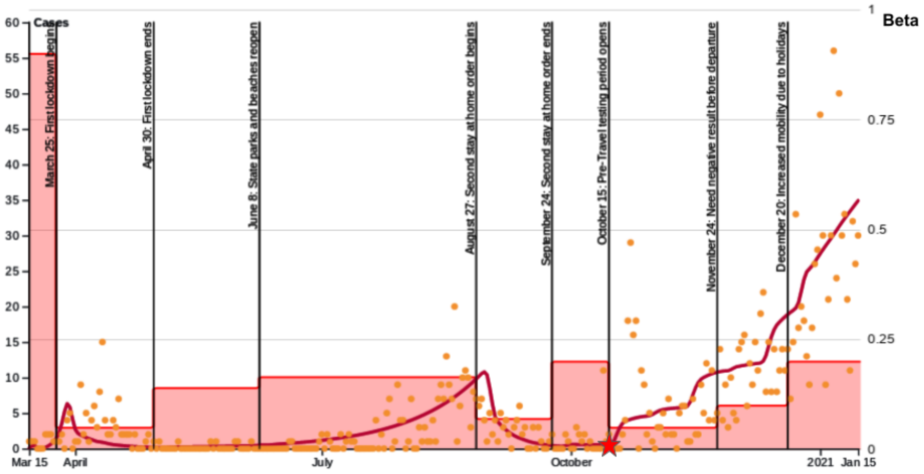

Fig 3. Maui county: COVID-19 daily count and key events reflecting a change in behavior.

The four zip codes with the largest counts for Maui county can be found in 6

Kauaʻi County

Due to the low numbers on Kauaʻi a model fit using our compartmental model could not be achieved. It can be observed on Fig.4 that the daily cases started following an exponential growth, it was attributed to travelers which prompted the mayor of Kauaʻi to request authorization to opt-out from the safe travel program. It was followed by a decrease in

Transmission rates		
Mar 15 - Mar 24	Mar 25 - Apr 29	Apr 30 - Jun 7
$\beta = 0.90$	$\beta = 0.05$	$\beta = 0.14$
Jun 8 - Aug 26	Aug 27 - Sep 23	Sep 24 - Oct 14
$\beta = 0.165$	$\beta = 0.07$	$\beta = 0.20$
Oct 15 - Nov 23	Nov 24 - Dec 19	Dec 20 - Jan 15
$\beta = 0.05$	$\beta = 0.10$	$\beta = 0.2$

Table 5. Optimized transmission rates to fit Maui county data. They reflect the State and Maui non-pharmaceutical mitigation measures.

Maui County Zip code	Population Estimate	Cumulative Daily cases	Cum. Daily cases per 100 inhabitants
96732	29075	278	9.5
96753	28737	259	9
96761	22301	240	11
96768	18529	90	5
96793	34036	211	6

Table 6. The four zip codes with the largest cumulative distribution of daily cases.

numbers and stabilization. A new peak can be observed right after the safe travel program was authorized reinstated by Kauaʻi for intercounty travelers. The numbers are so small that is it extremely difficult to draw any additional conclusion.

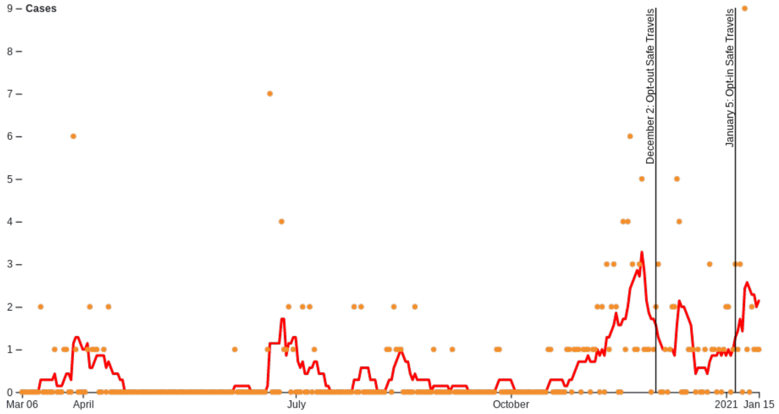

Fig 4. Kauaʻi county: COVID-19 daily count (orange) and 7-day average (red). The Opt-in Safe Travel on Jan 5 is only for intercounty travelers.
